# DNA barcode reference library of bush-crickets (Orthoptera, Tettigoniidae) from the Iberian Peninsula

**DOI:** 10.1038/s41598-025-06695-2

**Published:** 2025-09-30

**Authors:** Jorge Gutiérrez-Rodríguez, Alejandro Zaldívar-Riverón, E. Karen López-Estrada, Pablo Barranco, Mario García-París

**Affiliations:** 1 School of Life and Environmental Sciences, University of Sydney, Sydney, NSW Australia; 2 Department of Biodiversity and Evolutionary Biology, Museo Nacional de Ciencias Naturales (MNCN-CSIC), Madrid, Spain; 3 Instituto de Biología, Colección Nacional de Insectos, Universidad Nacional Autónoma de México, Ciudad de México, Mexico; 4 Unidad de Síntesis en Sistemática y Evolución (UniSSE), Instituto de Biología, Universidad Nacional Autónoma de México, Ciudad de México, Mexico; 5 Departamento de Biología y Geología, CITE-IIB, CECOUAL, Universidad de Almería, Ctra. Sacramento, s/n, La Cañada, Almería, 04120 Spain

**Keywords:** Cytochrome *c* oxidase subunit I (cox1), Introgression, Incomplete lineage sorting, Hybridization, Iberian Peninsula, Katydids, Entomology, DNA sequencing

## Abstract

Curated DNA barcode reference libraries are crucial for advancing environmental DNA (eDNA) studies, monitoring biological invasions, reliable biodiversity assessments, accurate species identification, etc. However, DNA barcode databases remain highly incomplete for most invertebrate taxa. In this study, we present the most comprehensive reference library to date for the family Tettigoniidae (Orthoptera) from the Iberian Peninsula—the most species-rich orthopteran family globally, with over 8,000 valid species. We generated 402 new DNA barcodes from at least 121 tettigoniid species from the Iberian Peninsula and integrated these with 169 previously published sequences. The resulting dataset comprises 571 barcoded specimens, representing 49 genera and 123 species, including many recently described taxa. Notably, we provide DNA barcodes for at least 68 described species that previously lacked them. Our dataset covers 85% of the tettigoniid species in the Iberian Peninsula and approximately 25% of European bush-cricket species. Furthermore, our analyses show that most tettigoniid species (95%) can be reliably identified using DNA barcoding. However, mitochondrial introgression events were detected in several species of the subfamilies Bradyporinae and Tettigoniinae, highlighting the need for cautious application of this molecular identification tool.

**Supplementary Information:**

The online version contains supplementary material available at 10.1038/s41598-025-06695-2.

## Introduction

Since the introduction of the DNA barcoding concept proposed by Hebert et al.^1^ as a global species identification system, the Barcode of Life Data Systems (BOLD^2^) has experienced exponential growth. For animals, this tool primarily relies on a specific region of the mitochondrial cytochrome *c* oxidase subunit I (cox1) gene, often referred as “DNA barcode”. Currently, the BOLD database (last accessed date: 9 April 2025) includes over 21,924,433 DNA barcodes, representing 261,919 animal species.

Initially conceived as a DNA-based identification system to facilitate the discovery of new taxa —using Kimura 2- parameter (K2P) distance as a criterion for barcode assignment^3,4^−there have been isolated efforts to use DNA barcodes alone for the formal description of species^5,6^. However, this practice has faced considerably criticism^7,8^. Beyond its original scope, DNA has expanded its initial focus. The emergence of DNA metabarcoding, which integrates traditional DNA barcoding with massively high throughput sequencing^9^ has revolutionized the fields of ecology, evolution, and conservation^10^ by enabling the identification of multiple species within mixed samples (bulk DNA^11^).

Despite its significant progress, the animal DNA barcode database remains still incomplete for most taxa. The lack of reliable reference libraries for many invertebrate groups has been a growing concern^12^. Numerous DNA samples cannot be identified at the species level due to insufficient representation in barcode reference libraries for many taxa^13^. The reliability of DNA barcodes depends largely on accurate identifications. Misidentifications can lead to flawed hypotheses, highlighting the importance of curating these databases with the expertise of taxonomists who have acquired their skills through years of training^14^. Consequently, curated DNA barcode databases are extremely important for integrative taxonomic studies. However, several phenomena, such as introgression by hybridization, incomplete lineage sorting, heteroplasmy, or co-amplification of nuclear mitochondrial paralogs (NUMTs), can complicate species identification using DNA barcodes and mitochondrial DNA in general within Metazoa^15–17^.

With approximately 1,300 genera and over 8,300 recognized species globally, the family Tettigoniidae (*sensu* Ciglano et al.^18^ i.e., Phaneropteridae + Tettigoniidae *sensu* Hochkirch et al.^19^) is the most species rich family within the insect order Orthoptera^18^. Members of this family are distributed across all continents except Antarctica, with the highest diversity found in tropical and temperate regions. Over the last decade, various studies examined the phylogenetic relationships among the tettigoniid subfamilies^20–22^. These molecular studies revealed extensive paraphyly within the family at subfamily level, consistent with previous morphological studies carried out by Hebard^23^. It is likely that several morphological features currently used to delineate katydid subfamilies and tribes are convergent adaptations to similar ecomorphs^22^.

The BOLD database currently contains over 13,000 public DNA barcodes representing more than 1,000 species of Tettigoniidae worldwide. However, significant geographic persist, with most data originating from China, Costa Rica, and the U.S.A. By contrast, there is still a substantial gap in the DNA barcode library for the European tettigoniid species, even though the continent hosts more than 500 species, with its highest species richness found along the Mediterranean climate region and the Balkan Mountain range. Currently, most of the available tettigoniid DNA barcodes for Europe come from Central Europe^16^ the Balkan Peninsula^24,25^ and Portugal^26^.

In the Iberian Peninsula, the Tettigoniidae are represented by more than 140 species across 51 genera. Consequently, this region is recognized as one of the most biodiverse areas for this taxon in Europe, characterized by high levels of endemism in Tettigoniidae, with approximately 65% of species being endemic (own elaboration). Factors such as land-bridges between African and European continents, the region’s mountainous topography providing multiple refugia, and the climate fluctuations of the Pleistocene have driven high levels of species endemism^27^. The Iberian Peninsula serves as the primary diversification center for several genera of flightless bush-crickets, in particular for the genera *Neocallicrania*, *Pycnogaster*, and *Ctenodecticus*^28,29^.

Some species occupy highly restricted areas and are known only from a few localities, such as *Coracinotus squamiferus*^30^ or *Roeseliana oporina*^31,32^. Moreover, the taxonomic status of many species is uncertain mainly because they are known only from a handful of specimens, such as the saddle bush-crickets^19^ (subfam. Bradyporinae). Since the beginning of the twenty-first century, approximately 20 new tettigonid species have been described from the Iberian Peninsula, most of which have very restricted distributions, including *Lluciapomaresius eclipticus*^33^ *Neocallicrania barrosi*^34^ and *Baratia sari*^35^.

In this study, we present the most comprehensive DNA barcoding reference library for the Tettigoniidae from the Iberian Peninsula, covering 85% of its recognized species, and including numerous endemic species. This library represents ca. 25% of European bush-cricket species and includes several taxa classified as Endangered or Data Deficient by the IUCN^19^.

## Results

**Fig. 1:**
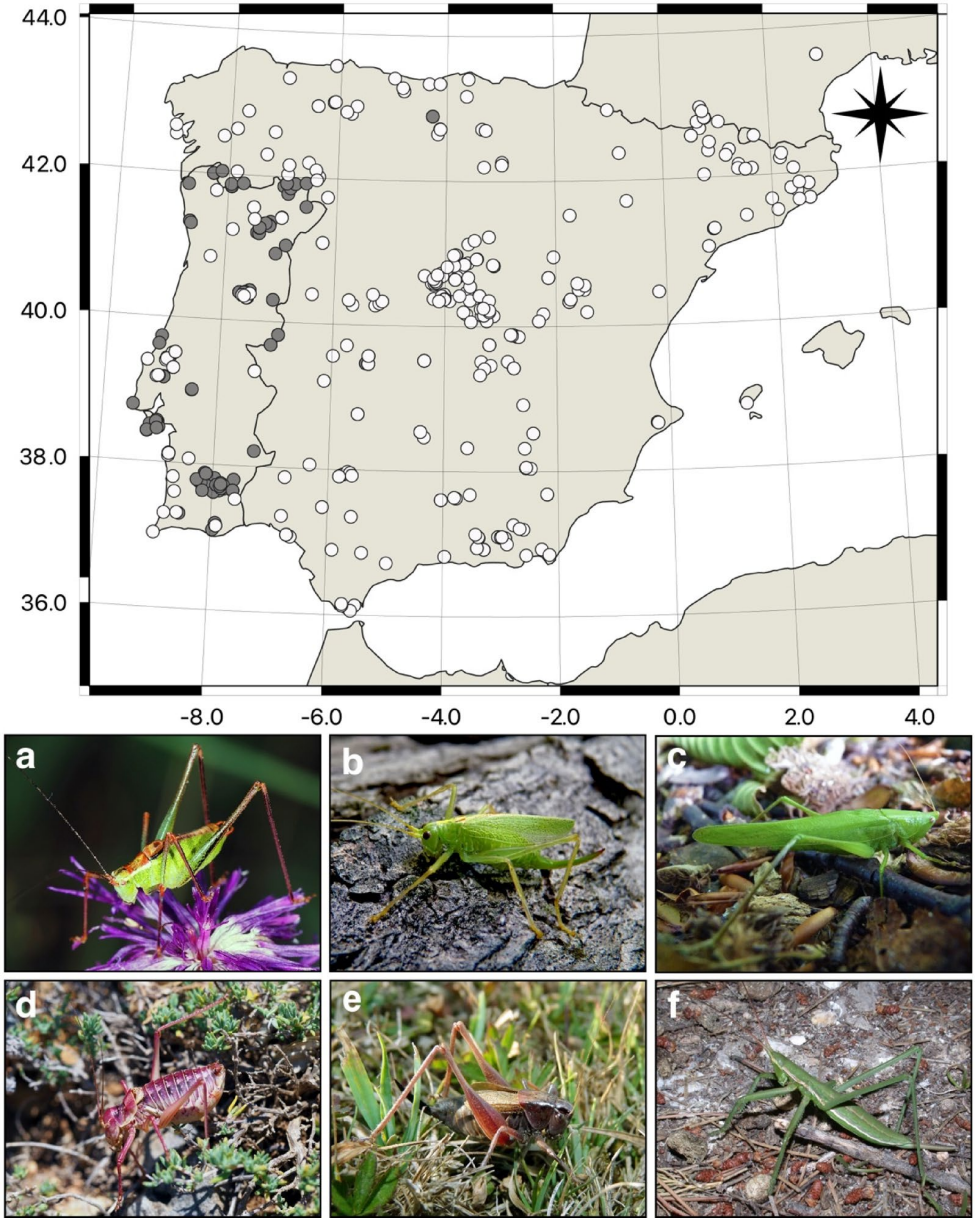
Map of sampling locations in Iberian Peninsula. White dots represent newly generated DNA barcodes, while black dots indicate previously published DNA barcodes. Coordinates are provided in Table S1. (**a**–**f**) Representative species from each Tettigoniidae subfamily; (**a**) *Leptophyes punctatissima* (subfam. Phaneropterinae); (**b**) *Meconema thalassium* (subfam. Meconematinae); (**c**) *Ruspolia nitidula* (subfam. Conocephalinae); (**d**) *Metrioptera maritima* (subfam. Tettigoniinae); (**e**) *Ephippigerida asella* (subfam. Bradyporinae); (**f**) *Saga pedo* (subfam. Saginae).

**Fig. 2:**
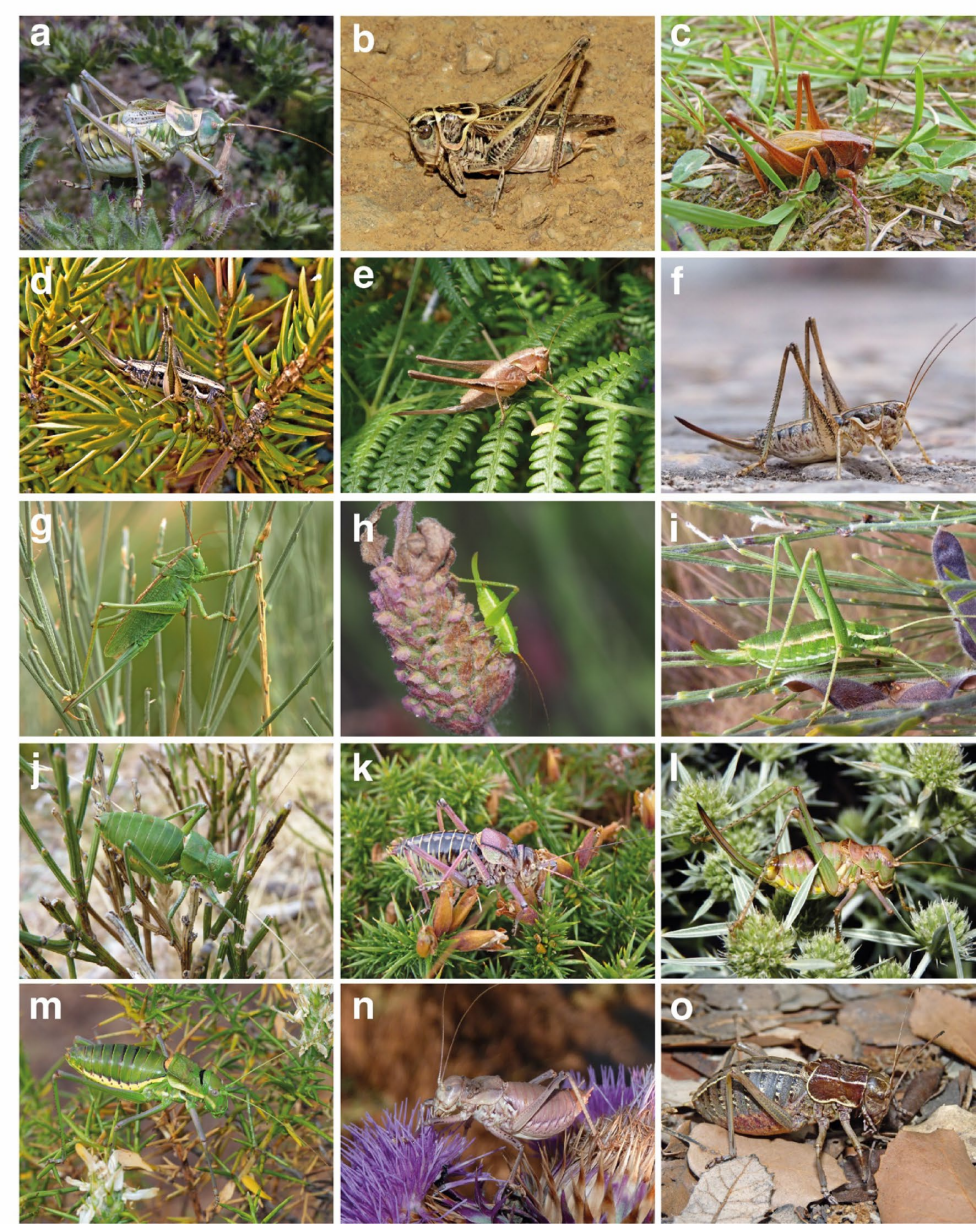
Representative specimens of endemic bush-crickets from the Iberian Peninsula. (**a**) *Amphiestris baetica* (Sevilla, Salteras); (**b**) *Montana carpetana* (Badajoz, Castuera); (**c**) *Zeuneriana burriana* (Asturias, Cabrales); (**d**) *Ctenodecticus lusitanicus* (Guarda, Seia); (**e**) *Antaxius florezi* (Braga, Terras de Bouro); (**f**) *Pterolepis cordubensis* (Cordoba, Carmona); (**g**) *Tettigonia hispanica* (Ávila, Hoyos del Espino); (**h**) *Cyrtaspis tuberculata* (Huelva, Almonte); (**i**) *Odontura macphersoni* (Guarda, Manteigas); (**j**) *Callicrania demandae* (La Rioja, Valdezcaray); (**k**) *Neocallicrania bolivarii* (Asturias, Villanueva de Oscos); (**l**) *Ephippigerida carinata* (Cuenca, Fuentenava de Jábaga); (**m**) *Steropleurus recticarinatus* (Huelva, Almonte); (**n**) *Platystolus surcularius* (Ciudad Real, Campo de Criptana); (**o**) *Pycnogaster gaditana* (Cadiz, Jerez de la Frontera).

**Fig. 3:**
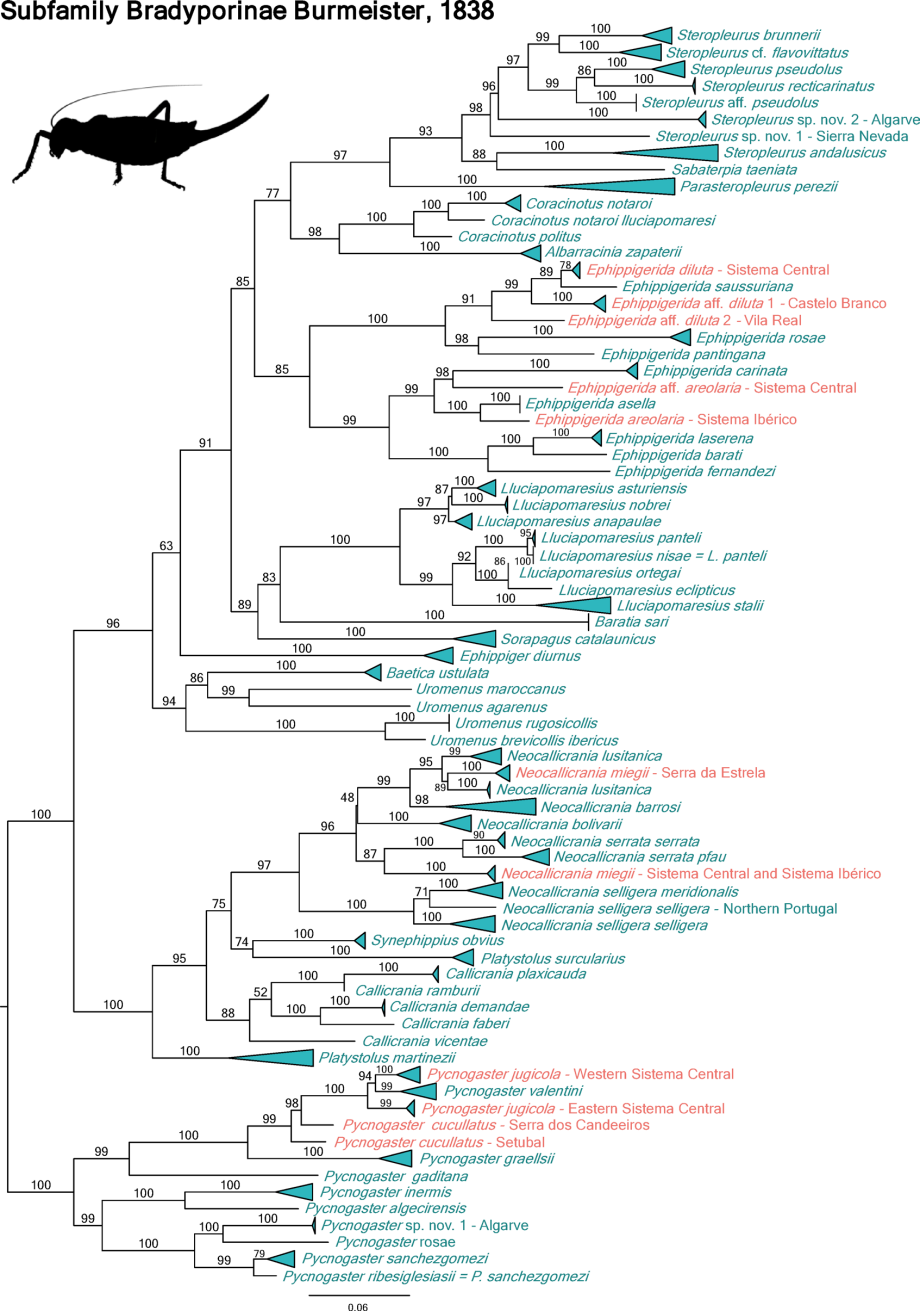
Maximum likelihood (ML) topology based on cox1 sequences for the subfamily Bradyporinae. Triangle width shows the relative number of sequence divergence; zero divergence is represented by a straight line. Bootstrap support values are shown above branches. Species with multiple populations that are not monophyletic are highlighted in red.

A total of 402 bush-cricket specimens were successfully sequenced for the cox1 gene, complemented by an additional 169 sequences from previous studies^26,36^ (Fig. 1), as listed in Table S1. The sequences were morphologically assigned to at least 123 species, spanning six tettigoniid subfamilies and 49 genera present in the Iberian Peninsula (Table 1), representing 85% of the species recorded in the region (Fig. 2). Among these 123 species, we generated DNA barcodes for at least 68 described species (55.3%) that previously lacked barcode representation. We also identified at least three putative new species (Fig. 3). The DNA barcode lengths ranged from 599 to 658 base pairs (bp). The number of specimens sequenced per species varied from 1 to 26 (Table S1), with an average of 3.26 barcodes per species.

**Table 1:** Number of valid species reported for each subfamily of Tettigoniidae in the Iberian Peninsula. Number and percentage of species from each subfamily included in this study. Finally, number of DNA barcodes included per subfamily.

Iberian Tettigoniidae subfamilies	No. of valid species	No. of included species	% of species	No. of DNA barcodes
Bradyporinae	67	58	86.57	193
Conocephalinae	5	3	60	22
Meconematinae	6	4	66.67	10
Phaneropterinae	15	11	73.33	60
Saginae	1	1	100	2
Tettigoniinae	50	46	92	284

The Bradyporinae emerged as the most species rich tettigoniid subfamily, with specimens sequenced from at least 58 species (Table 1), including several taxa likely new to science (Fig. 3). Unfortunately, it was not possible to generate DNA barcodes from the following nine species of the Bradyporinae: *Callicrania belarrensis*, *Ca. denticulata*, *Coracinotus squamiferus*, *Ephippigerida longicauda*, *Sabaterpia hispanica*, *Sa. paulinoi*, *Steropleurus castellanus*, *St. obsoletus*, and *Parasteropleurus martorellii*.

We successfully generated DNA barcodes for most of the recognized species of Conocephalinae from the Iberian Peninsula (Table 1), except for *Conocephalus dorsalis* and *Co. concolor hispanicus*. Similarly, we sequenced all but two species of Meconematinae (Table 1), with *Canariola quinonesi*, and *Meconema meridionale* being the only missing taxa. We also obtained DNA barcodes sequences for *Saga pedo*, the only representative species of Saginae in the Iberian Peninsula, generating two sequences for this species. Within Phaneropterinae, we generated DNA barcodes of 73.33% (11 out of 15; Table 1) of the recognized species in the region. However, specimens of *Barbitistes serricauda*, *Phaneroptera laticerca*, *Polysarcus denticauda*, and *Po. scutatus* were not available.

**Fig. 4:**
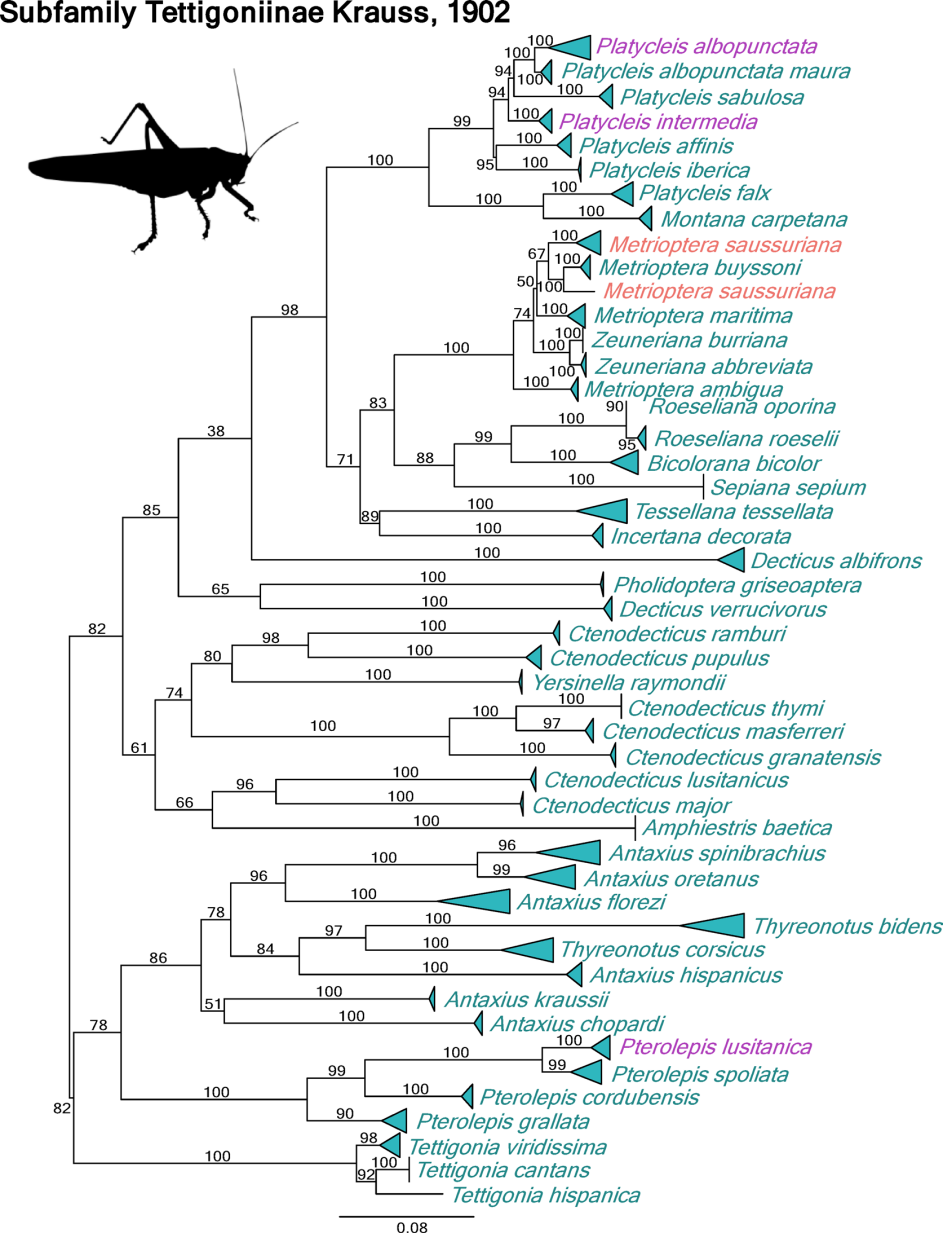
Maximum likelihood (ML) phylogenetic tree based on Cox1 sequences for the subfamily Tettigoniinae. Triangle width shows the relative number of sequence divergence; zero divergence is represented by a straight line. Bootstrap support values are shown above branches. Red color highlights species comprising of multiple populations that are not monophyletic. Species with multiple populations that are not monophyletic are highlighted in red, and clades grouping sequences from multiple species due to introgression events are shown in purple.

Finally, we sequenced 46 (92%; Table 1) of the 50 recognized species of Tettigoniinae from the Iberian Peninsula, the second species rich subfamily in this region (Fig. 4). Specimens of *Gampsocleis glabra*, *Metrioptera brachyptera*, *Antaxius tavaresi*, and *Rhacocleis annulata* (alien species) could not be collected.

### ML trees

The DNA barcodes showed high congruence with morphology-based identifications, as sequences for each species typically formed exclusive clades in the ML analyses (Figs. 3 and 4 and suppl. Figs. S1, S2 and S3). We identified 95% of the species (117 species) to the species level using DNA barcoding, with molecular assignments being congruent with morphological identifications. Only six species could not be confidently assigned to a species level using DNA barcoding methods. However, we discarded two sequences due to their incongruent phylogenetic placement in the phylogenetic tree, most likely as a result of NUMTs in *Ephippiger diurnus*. Within the dataset, no stop codons were found.

We did not recover the monophyly of some genera and recovered apparently incorrect phylogenetic relationships among several taxa. In certain cases, short fragments of cox1 produced phylogenetic reconstructions that, despite showing relatively high bootstrap values, were inaccurate. For instance, *Antaxius hispanicus* was found within the clade containing the two *Thyreonotus* species, and another clade grouped *Amphiestris baetica* with *Ctenodecticus major* and *Ct. lusitanicus*.

We uncovered previously unnoticed genetic diversity within several genera, primarily in Bradyporinae, identifying multiple independent lineages in widely distributed species belonging to the genera *Steropleurus*,* Ephippigerida*, and *Pycnogaster*. Within *Pycnogaster*, we detected a novel lineage closely related to *Py. rosae*. In contrast, populations of *Ephippigerida areolaria* from the Sistema Central Mountain range formed a clade with *Ep. carinata*, which appeared as the sister group to a clade comprising other *Ep. areolaria* samples from the Sistema Iberico and *Ep. asella*.

We observed some incongruence between mitochondrial and morphological characters in species from the subfamilies Bradyporinae and Tettigoniinae (Figs. 3 and 4). Within Bradyporinae, one specimen from the type locality of *Lluciapomaresius nobrei* clustered with *Ll. stalii*. Additionally, specimen clusters of *Neocallicrania miegii* were not monophyletic, as those from the Serra da Estrela formed a clade with populations of *Ne. lusitanica*. For *Pycnogaster cucullatus*, sequences did not form a monophyletic group, most likely revealing evidence of introgression from *Py. jugicola* in one population.

In the subfamily Tettigoniinae, several instances of incongruence were detected. Specimens from the western distribution of *Pterolepis spoliata* clustered with *Pt. lusitanica*. Within the genus *Platycleis*, numerous specimens morphologically identified as *Pl. sabulosa* formed a monophyletic group with *Pl. albopunctata*. Populations identified as *Pl. sabulosa* included specimens from both mitochondrial groups without displaying a consistent geographical pattern. Furthermore, specimens of *Pl. albopunctata* from the northern Iberian Peninsula were grouped with *Pl. intermedia*. Similarly, in *Metrioptera saussuriana*, a specimen from the Pyrenees formed a monophyletic group with *Me. buyssoni*.

### Genetic distances

**Fig. 5:**
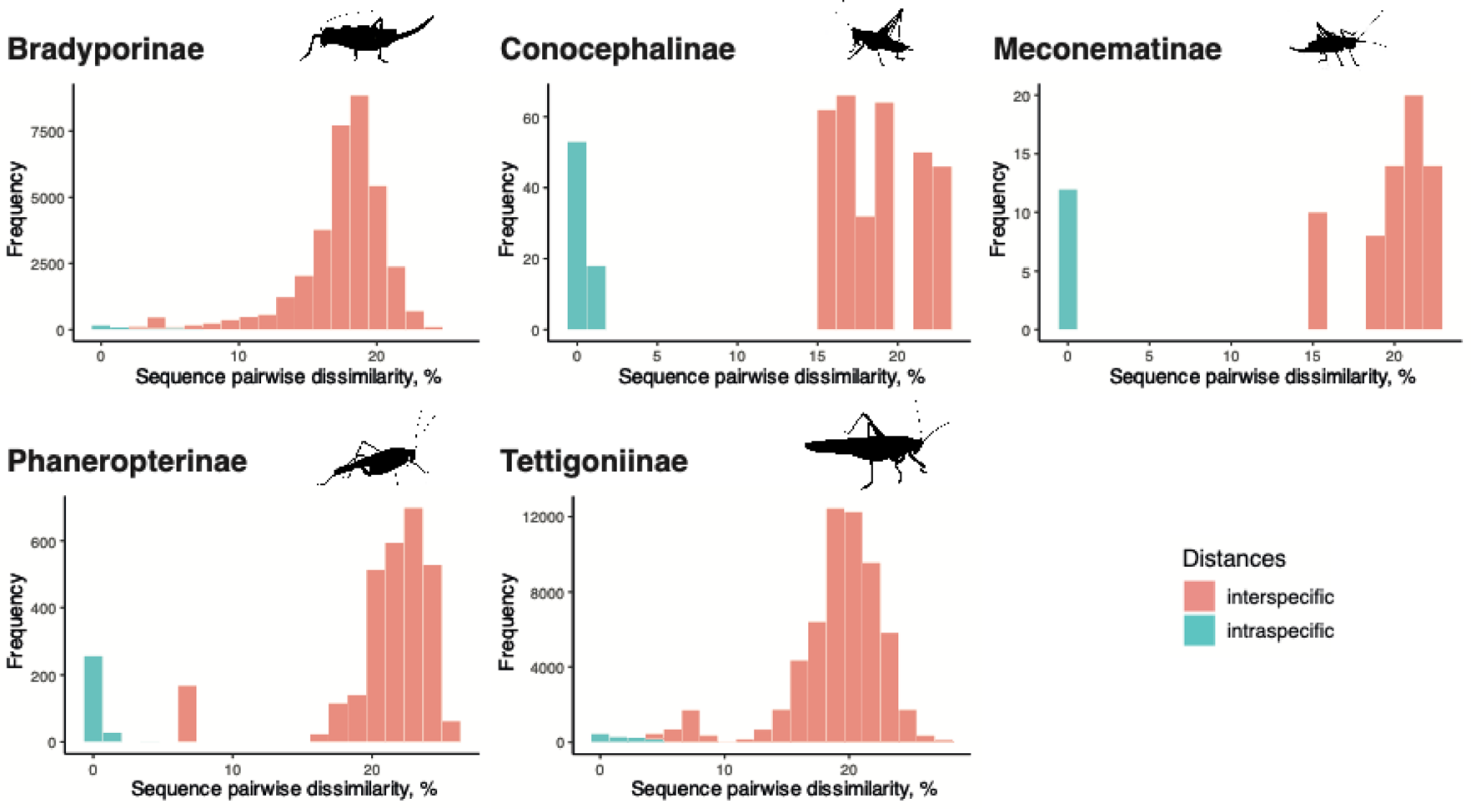
Intra- and interspecific genetic distances within each subfamily.

**Fig. 6:**
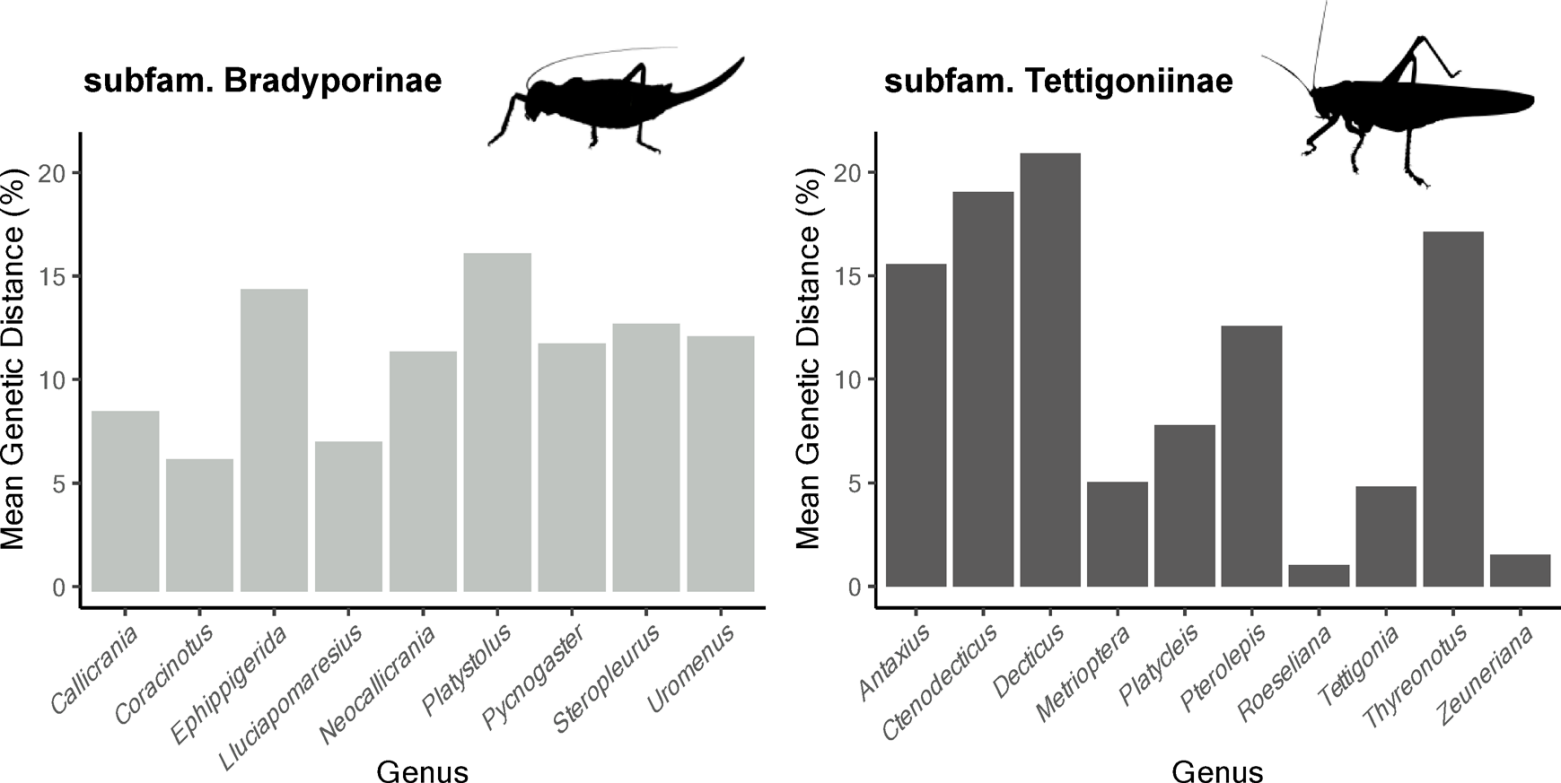
Mean interspecific genetic distances within each genus for the subfamilies Tettigoniinae and Bradyporinae.

We assessed the maximum intraspecific variability within the tettigoniid subfamilies (Fig. 5). The results revealed the following maximum intraspecific distances: 9.51% in Bradyporinae, 1.30% in Conocephalinae, 0.31% in Meconematinae, 5.37% in Tettigoniinae, and 5.31% in Phaneropterinae. Additionally, we calculated the minimum interspecific genetic distances from each subfamily, which were as follows: 2.15% in Bradyporinae, 15.66% in Conocephalinae, 15.26% in Meconematinae, 0.90% in Tettigoniinae, and 6.52% in Phaneropterinae. The minimum interspecific distance within Tettigoniinae was 0.90%, observed in the pairwise comparison between *Roeselina oporina* and *Ro. roeselii*. Unusually high intraspecific distances (> 5%) were detected in *Parasteropleurus perezii* and *Steropleurus andalusicus*. Finally, the mean interspecific genetic distances within each genus of Bradyporinae were relatively similar (6.35–16.27), whereas greater variability were found between genera in Tettigoniinae (1.01–20.91) (Fig. 6).

We also calculated intra- and intergeneric variability within the tettigoniid subfamilies (Suppl. Fig. S4). The maximum intrageneric distance and minimum intergeneric distance were: 20.47% and 9.20% in Bradyporinae; 16.46% and 18.11% in Conocephalinae; 15.65% and 15.66% in Meconematinae; 22.56% and 3.29% in Tettigoniinae; 25.10% and 16.69% in Phaneropterinae.

## Discussion

In this study, we present the most comprehensive DNA barcode reference library for the family Tettigoniidae in Europe, addressing significant gaps in the species catalogue at the European level. We conducted a thorough and detailed morphological identification of all specimens, identifying almost all collected specimens to species level. The taxonomic accuracy of the reference database is crucial, as it directly influences the interpretation and outcomes of ecological community studies^37^. Thus, DNA barcode reference libraries should be prioritized in studies that utilize environmental DNA and other metabarcoding approaches^38^.

Our results demonstrate the utility and potential of DNA barcoding for identifying species within Tettigoniidae in the Iberian Peninsula. Of the 123 described species included in this study, 117 species (95%) could be reliably identified using DNA barcodes, while six species could not be reliably identified using this method. The DNA barcodes generated here cover 85% of the species of this family reported for the Iberian Peninsula, and nearly 25% of European species. This represents a significant progress in the development of high-quality, publicly accessible reference databases for this ecologically important group.

Our results show a high congruence between morphology-based identifications and DNA barcodes, with most species forming exclusive monophyletic groups in the ML analyses (Figs. 3 and 4 and suppl. Figs. S1, S2 and S3). However, we encountered several challenges within the DNA barcoding identification system, leading to occasional inconsistencies in taxonomic assignments. Specifically, we observed incongruences between morphological characters and DNA barcodes in various subfamilies (notably in Bradyporinae and Tettigoniinae), where conspecific individuals failed to form a cluster (Figs. 3 and 4). These discrepancies may be the result of incomplete lineage sorting (ILS) due to ancestral polymorphism, contemporary or historical hybridization leading to mitochondrial introgression^39,40^ or even *Wolbachia* infections^16^. However, the analysis of a single mitochondrial marker does not allow us to discern the responsible mechanisms responsible for these incongruences^41^. Nevertheless, the observed geographic patterns of incongruence suggest that different evolutionary processes might be driving discordant results. In some species, these phenomena are restricted to a population or geographical area. For example, specimens of *Pt. spoliata* from the western Iberian Peninsula were grouped with *Pt. lusitanica*, and specimens of *Ll. nobrei* from the Serra da Estrela which were grouped with *Ll. stali*. Similarly, in *Me. saussuriana*, a specimen from the Pyrenees, located near the distribution range of *Me. buyssoni*, formed a monophyletic group with specimens of the latter, strongly suggesting a potential contact zone between the two species.

In the genus *Platycleis*, specimens of *Pl. sabulosa* were inconsistently grouped, either a species-specific monophyletic cluster or clustering together with specimens of *Pl. albopunctata*. Such patterns may result from processes like mitochondrial introgression by hybridization, which have been widely reported in other orthopteran families in the past^16,42,43^. Moreover, in groups such as Acrididae, the low efficacy of DNA barcoding has been linked to incomplete lineage sorting processes^17^.

Despite the inherent limitations of using a single molecular marker, this approach enables an initial assessment of genetic diversity across various taxonomic groups. Consistent with prior DNA barcoding studies of Tettigoniidae^44–46^ our results reveal hidden genetic diversity across the Iberian Peninsula. Furthermore, they give evidence for the existence of undescribed species of katydids and cryptic lineages within currently recognized species that have previously gone unnoticed. For example, unexpected genetic diversity was detected within the genera *Ephippigerida*,* Steropleurus*, and *Pycnogaster* (Fig. 3).

Intra- and interspecific genetic distances revealed significant variability within the Tettigoniidae subfamilies (Fig. 5, Table S2). The maximum intraspecific genetic distance was particularly high in Bradyporinae (8.79%), while the minimum interspecific genetic distance was strikingly low in some groups within Tettigoniinae. For instance, the close genetic relationship between morphologically well-defined species from the genera *Metrioptera*,* Roeseliana* and *Platycleis*—such as *Ro. oporina* and *Ro. roeselii* (0.90%)—is especially noteworthy. The high intraspecific cox1 distances observed in Bradyporinae may be associated with the flightlessness and limited dispersal ability of its species. This limited dispersal capability could have enabled the formation of microrefugial due to ecological fragmentation, leading to genetic isolation and the emergence of divergent allopatric lineages^47^.

Our findings are consistent with the estimates reported for other Orthoptera genera, where interspecific divergences typically range from 7 to 15%, and intraspecific distances remain below 5%^48^. It is important to highlight the genera within Tettigoniinae that belong to the *Metrioptera - Platycleis* group (Palearctic Platycleidini with unarmed prosternum^49^)—including the aforementioned genera as well as *Zeuneriana* and *Roeseliana* (Fig. 6). These genera display limited interspecific divergence, with intrageneric mean distances ranging from 1.02 to 7.79%. These values contrast sharply with those observed in various genera with diversification centers in the Iberian Peninsula, such as *Ctenodecticus* (19.04%) and *Antaxius* (15.56%). The contrasting evolutionary histories of these taxa—likely driven by a recent diversification process within the *Metrioptera - Platycleis* group across the Western Palearctic—merit further exploration in future evolutionary studies.

At European level, the Tettigoniidae include a high proportion of threatened orthopteran species, alongside Pamphagidae and Rhaphidophoridae, due to the fact that many species are flightless and have small distribution ranges^19^. In this study, we provided DNA barcodes for two critical endangered species (CR: *Ctenodecticus major* and *Platycleis iberica*), six endangered species (EN: *Amphiestris baetica*; *Baetica ustulata*; *Ctenodecticus lusitanicus*; *Ephippigerida asella*; *Ephippigerida rosae*; *Metrioptera buyssoni*), and three vulnerable species (VU: *Coracinotus notarioi*; *Platycleis falx*; *Roeseliana oporina*).

At least half of the approximately 20 tettigoniid species from the Iberian Peninsula that are missing in this study are known only from a handful of specimens, most of which were collected decades ago. The taxonomic status of some of these species is still unclear, as is the case for *Ephippigerida longicauda* or *Antaxius tavaresi*. Nevertheless, these species are primarily categorized as Data Deficient (DD) by the IUCN^19^. Unfortunately, species classified as DD are often excluded from conservation and management policies. Predictions suggest that more than half of DD species may be threatened with extinction^50,51^.

Despite the progress achieved, there are still evident taxonomic gaps in various tettigonid taxa, particularly in less-studied genera such as *Steropleurus*, *Ephippigerida* or *Pycnogaster*. Specifically, our molecular analyses revealed unexpected high diversity within genus *Steropleurus* (Fig. 3). Previous work by Barat^52^ had already emphasized the need for detailed studies to clarify the identity of species such as *St. brunnerii*, *St. castellanus*, and *St. flavovittatus*. However, our study also uncovered significant diversity within the genus in the southern Iberian Peninsula.

This study makes a substantial contribution to the reference library of DNA barcodes for Tettigoniidae, providing valuable data for the identification and conservation of these species in the Iberian Peninsula and Southern Europe. Future research should aim to expand the geographical scope of this taxonomic integrative effort and include intraspecific variability across all species.

## Materials and methods

### Study area and sampling methods

Sampling was conducted exclusively on adults across the Iberian Peninsula through various scientific surveys carried out between spring and autumn from 2001 to 2024.

A total of 402 specimens were collected, representing at least 121 species (Fig. 1 and Table S1). The specimens were preserved in absolute ethanol. Species determination was carried out following the relevant literature^53,54^. Published records of 144 currently recognized Tettigoniidae species from the Iberian Peninsula were reviewed, although some taxa remain taxonomically uncertain since they are known only from few specimens or have a very restricted distribution (e.g.,* Steropleurus castellanus* and *Ephippigerida longicauda*^52^). Recent taxonomic updates were incorporated, including newly described species (e.g., *Ephippigerida fernandezi and Pycnogaster rosae*) and those synonymized (e.g., *Lluciapomaresius nisae* considered a junior synonym of *Ll. panteli*^55^ and *Pycnogaster ribesiglesiasii*, considered a junior synonym of *Py. sanchezgomezi*^56^).

### DNA extraction, amplification, sequencing and alignment

Genomic DNA was extracted from the hind leg tissue of each specimen using the NucleoSpin Tissue- Kit (Macherey-Nagel), following the manufacturer’s protocol. The most widely used DNA barcode locus for animals sensu Hebert et al. (2003), representing a 658 bp fragment of the cox1 gene^3^was amplified. The following primer pairs were used: LCO1490^57^/COI-H^58^ and Orthoptera-specific J/Orthoptera-specific N^59^. Additionally, a custom primer pair was designed for the genus *Platycleis* due to we amplification issues with the standard primers: JGR-COIL 5′ – ATTTTTGGAGCTTGAGCAGGT – 3’ and JGR-COIH 5′ – GAAAAGGTGTTGGTATAAAATAGGG – 3’) (599 bp). PCR reactions were performed in a 25 µl volume containing 2 µl of DNA, 1 unit of Taq Polymerase (Biotools), 0.2 µM of each primer, 0.4 mM dNTP, 1 mM MgCl_2_, and 2.5 µl of of buffer containing 2 mM MgCl_2_. PCR products were precipitated with sodium acetate and ethanol and then resuspended in 20 µl of ultrapure water. The PCR products were directly sequenced at Macrogen Inc. (Macrogen Europe, Madrid, Spain).

Chromatograms were edited and aligned with Geneious Prime v 2022.0.1 (Biomatters, Auckland, New Zealand). The alignments were translated to amino acids to detect potential NUMTs. Sequences were deposited in BOLD under the project “DNA barcode reference library of Iberian bush-crickets” (dx.doi.org/10.5883/DS-23034539) and in GenBank (Accession numbers are provided in Table S1). The alignment also includes the previously published reference barcoding library of Orthoptera from continental Portugal^26^ as well as sequences from the recently described *Antaxius oretanus*^36^.

A maximum-likelihood (ML) phylogenetic tree was built for each subfamily using the program IQ-TREE2 v2.5.7^60^, except for the subfamily Saginae, which contains only one species in the Iberian Peninsula. To select the best-fit substitution model of each matrix, the Bayesian Information Criterion (BIC) was applied using ModelFinder^61^. We performed 10,000 ultrafast bootstrap replicates^62^ to estimate branch support bootstrap values.

Intra- and interspecific genetic distances within each subfamily were estimated with the Kimura 2-parameter (K2P) evolutionary model^63^ implemented in the R package Ape v5.7-1^64^, following the protocol of Alexander Ordynets (10.17504/protocols.io.n8jdhun). We also calculated intra- and intergeneric genetic distances within each subfamily. Introgressed sequences and candidate *NUMTs* were excluded from all genetic distance analysis. We also calculated genetic distances within each genus for the most the most specious subfamilies, namely Bradyporinae and Tettigoniinae.

## Electronic supplementary material

Below is the link to the electronic supplementary material.

Supplementary Material 1

## Data Availability

The newly obtained sequences from this study have been deposited in GenBank under the primary accession code SUB14828494 and in BOLD within the project titled “DNA barcode reference library of Iberian bush-crickets” (dx.doi.org/10.5883/DS-23034539). Alignments and phylogeny trees generated during the current study have been deposited at Figshare repository (https://doi.org/10.6084/m9.figshare.28156892).

## References

[1] HebertP.D., Cywinska, A., Ball, S. L. & DeWaard, J. R. Biological identifications through DNA barcodes. *Proc. Roy Soc. Lond. Ser. B Biol. Sci.* **270**, 313–321. 10.1098/rspb.2002.2218 (2003). 10.1098/rspb.2002.2218 PMC1691236 12614582

[2] Ratnasingham, S., Hebert, P. D. & BOLD The barcode of life data system. *Mol. Ecol. Notes*. **7**, 355–364. 10.1111/j.1471-8286.2007.01678.x (2007). http://www.barcodinglife.org 18784790 10.1111/j.1471-8286.2007.01678.x PMC1890991

[3] Hebert, P. D., Ratnasingham, S. & De Waard, J. R. Barcoding animal life: cytochrome c oxidase subunit 1 divergences among closely related species. *Proc. Roy Soc. Lond. Ser. B Biol. Sci.* **270**, S96–S99. 10.1098/rsbl.2003.0025 (2003). 10.1098/rsbl.2003.0025 PMC1698023 12952648

[4] Ratnasingham, S. & Hebert, P. D. A DNA-based registry for all animal species: the barcode index number (BIN) system. *PLoS One*. **8**, e66213. 10.1371/journal.pone.0066213 (2013). 23861743 10.1371/journal.pone.0066213 PMC3704603

[5] Meierotto, S. et al. A revolutionary protocol to describe understudied hyperdiverse taxa and overcome the taxonomic impediment. *Deut Entomol. Z.* **66**, 119–145. 10.3897/dez.66.34683 (2019).

[6] Sharkey, M. J. Minimalist revision and description of 403 new species in 11 subfamilies of Costa Rican braconid parasitoid wasps, including host records for 219 species. *ZooKeys* **1013**, 1–665. 10.3897/zookeys.1013.55600 (2021). 34512087 10.3897/zookeys.1013.55600 PMC8390796

[7] Zamani, A., Vahtera, V., Sääksjärvi, I. E. & Scherz, M. D. The omission of critical data in the pursuit of ‘revolutionary’methods to accelerate the description of species. *Syst. Entomol.* **46**, 1–4. 10.1111/syen.12444 (2021).

[8] Zamani, A. DNA barcodes on their own are not enough to describe a species. *Syst. Entomol.* **47**, 385–389. 10.1111/syen.12538 (2022).

[9] Coissac, E., Riaz, T. & Puillandre, N. Bioinformatic challenges for DNA metabarcoding of plants and animals. *Mol. Ecol.* **21**, 1834–1847. 10.1111/j.1365-294X.2012.05550.x (2012). 22486822 10.1111/j.1365-294X.2012.05550.x

[10] Gostel, M. R. & Kress, W. J. The expanding role of DNA barcodes: indispensable tools for ecology, evolution, and conservation. *Diversity* **14**, 213. 10.3390/d14030213 (2022).

[11] Liu, M., Clarke, L. J., Baker, S. C., Jordan, G. J. & Burridge, C. P. A practical guide to DNA metabarcoding for entomological ecologists. *Ecol. Entomol.* **45**, 373–385. 10.1111/een.12831 (2020).

[12] Santos, B. F. Enhancing DNA barcode reference libraries by harvesting terrestrial arthropods at the smithsonian’s National museum of natural history. *Biodivers. Data J.* **11**, e100904. 10.3897/BDJ.11.e100904 (2023). 38327288 10.3897/BDJ.11.e100904 PMC10848724

[13] Recuero, E., Etzler, F. E. & Caterino, M. S. Most soil and litter arthropods are unidentifiable based on current DNA barcode reference libraries. *Curr. Zool.* **70**, 637–646. 10.1093/cz/zoad051 (2024). 39463700 10.1093/cz/zoad051 PMC11502157

[14] Ekrem, T., Willassen, E. & Stur, E. A comprehensive DNA sequence library is essential for identification with DNA barcodes. *Mol. Phylogenet Evol.* **43**, 530–542. 10.1016/j.ympev.2006.11.021 (2007). 17208018 10.1016/j.ympev.2006.11.021

[15] Song, H., Moulton, M. J. & Whiting, M. F. Rampant nuclear insertion of MtDNA across diverse lineages within Orthoptera (Insecta). *PloS One*. **9**, e110508. 10.1371/journal.pone.0110508 (2014). 25333882 10.1371/journal.pone.0110508 PMC4204883

[16] Hawlitschek, O. et al. DNA barcoding of crickets, Katydids and grasshoppers (Orthoptera) from central Europe with focus on austria, Germany and Switzerland. *Mol. Ecol. Resour.* **17**, 1037–1053. 10.1111/1755-0998.12638 (2017). 27863033 10.1111/1755-0998.12638

[17] Nabholz, B. Incomplete lineage sorting explains the low performance of DNA barcoding in a radiation of four species of Western European grasshoppers (Orthoptera: acrididae: *Chorthippus*). *Biol. J. Linn. Soc.* **141**, 33–50. 10.1093/biolinnean/blad106 (2024).

[18] Cigliano, M. M., Braun, H., Eades, D. C. & Otte, D. Orthoptera species file. http://orthoptera.speciesfile.org. (Accessed 12 June 2024).

[19] Hochkirch, A. et al. European red list of grasshoppers, crickets and bush-crickets. (Publications Office of the European Union, 2016).

[20] Mugleston, J. D., Song, H. & Whiting, M. F. A century of paraphyly: A molecular phylogeny of Katydids (Orthoptera: Tettigoniidae) supports multiple origins of leaf-like wings. *Mol. Phylogenet Evol.* **69**, 1120–1134. 10.1016/j.ympev.2013.07.014 (2013). 23891949 10.1016/j.ympev.2013.07.014

[21] Mugleston, J. et al. Reinventing the leaf: multiple origins of leaf-like wings in Katydids (Orthoptera: Tettigoniidae). *Invertebr Syst.* **30**, 335–352. 10.1071/IS15055 (2016).

[22] Mugleston, J. D., Naegle, M., Song, H. & Whiting, M. F. A comprehensive phylogeny of Tettigoniidae (Orthoptera: Ensifera) reveals extensive ecomorph convergence and widespread taxonomic incongruence. *Insect Syst. Divers.* **2**, 5. 10.1093/isd/ixy010 (2018).

[23] Hebard, M. *Dermaptera and Orthoptera of Hawaii* (Bishop Museum Press, 1922).

[24] Chobanov, D. P., Kaya, S., Grzywacz, B., Warchałowska-Śliwa, E. & Çıplak, B. The Anatolio‐Balkan phylogeographic fault: A snapshot from the genus *Isophya* (Orthoptera, Tettigoniidae). *Zool. Scr.* **46**, 165–179. 10.1111/zsc.12194 (2017).

[25] Borissov, S. B., Heller, K. G., Çıplak, B. & Chobanov, D. P. Origin, evolution and systematics of the genus *Poecilimon* (Orthoptera: Tettigoniidae)—An outburst of diversification in the Aegean area. *Syst. Entomol.* **48**, 198–220. 10.1111/syen.12580 (2023).

[26] Pina, S. et al. The InBIO barcoding initiative database: DNA barcodes of Orthoptera from Portugal. *Biodivers. Data J.* **12**, e118010. 10.3897/BDJ.12.e118010 (2024). 38784157 10.3897/BDJ.12.e118010 PMC11112160

[27] Hewitt, G. M. Mediterranean peninsulas: the evolution of hotspots. In *Biodiversity Hotspots: Distribution and Protection of Conservation Priority Areas* (eds. Zachos, F. E. & Habel, J. C.) 123–147 (Springer, 2011).

[28] Kenyeres, Z., Rácz, I. A. & Varga, Z. Endemism hot spots, core areas and disjunctions in European Orthoptera. *Acta Zool. Cracov. Ser. B Invertebrata*. **52**, 189–211. 10.3409/azc.52b_1-2.189-211 (2009).

[29] Pfau, H. K. Untersuchungen Zur stridulation und phylogenie der Gattung *Pycnogaster* graells, 1851 (Orthoptera, tettigoniidae, Pycnogastrinae). *Mitt Schweiz. Entomol.* **61**, 167–183 (1988).

[30] Domenech-Fernández, M. Revisión preliminar Del Género *Coracinotus* barat, 2012 (Orthoptera: tettigoniidae: Bradyporinae). *Heteropterus Rev. Entomol.* **23**, 95–158 (2023).

[31] Gutiérrez-Rodríguez, J. & García-París, M. Rediscovery of the ghost bush-cricket *Roeseliana Oporina* (Orthoptera: Tettigoniidae) in central Spain. *J. Insect Conserv.* **20**, 149–154. 10.1007/s10841-016-9846-1 (2016).

[32] Díaz-Martínez, C., Rodríguez-Flores, P. C., Cardo-Maeso, N. & García-París, M. Distribución geográfica y amenazas Para La conservación Del Endemismo conquense *Roeseliana Oporina* (Orthoptera: Tettigoniidae). *Heteropterus Rev. Entomol.* **23**, 79–88 (2023).

[33] Barat, J. Descripción de *Steropleurus eclipticus* sp. n.(Othoptera: tettigoniidae: Ephippigerinae) Del sistema ibérico, Zaragoza (España). *Boletín De La. S E A*. **34**, 1–7 (2004).

[34] Barat, J. Revisión de la identidad de *Neocallicrania serrata* (Bolívar, 1885) y descripción de dos táxones afines: *Neocallicrania serrata pfaui* ssp. n. y *Neocallicrania barrosi* sp. n.(Orthoptera, Tettigoniidae, Bradyporinae, Ephippigerini). *Boletín de la S. E. A.* **52** 1–16 (2013).

[35] Llucià-Pomares, D. Descripción de Un Nuevo Género y especie de ephippigerini (Orthoptera: tettigoniidae: Bradyporinae) Del Pirineo central de Andorra (noreste Península ibérica). *Boletín De La. S E A*. **69**, 1–14 (2021).

[36] Gutiérrez-Rodríguez, J., Domenech-Fernández, M., Barranco, P. & García-París, M. Phylogeography and species distribution modeling unveil unnoticed pliocene diversity: the case of a montane Iberian bush-cricket, *Antaxius spinibrachius* (Orthoptera: Tettigoniidae). *Insect Syst. Divers.* **8**, 8. 10.1093/isd/ixae023 (2024).

[37] Bortolus, A. Error cascades in the biological sciences: the unwanted consequences of using bad taxonomy in ecology. *Ambio* **37**, 114–118. 10.1579/0044-7447(2008)37 (2008). [114:ECITBS]2.0.CO;2. 18488554 10.1579/0044-7447(2008)37[114:ecitbs]2.0.co;2

[38] Morinière, J. et al. A DNA barcode library for 5,200 German flies and midges (Insecta: Diptera) and its implications for metabarcoding-based biomonitoring. *Mol. Ecol. Resour.* **19**, 900–928. 10.1111/1755-0998.13022 (2019). 30977972 10.1111/1755-0998.13022 PMC6851627

[39] Degnan, J. H. & Rosenberg, N. A. Gene tree discordance, phylogenetic inference and the multispecies coalescent. *Trends Ecol. Evol.* **24**, 332–340. 10.1016/j.tree.2009.01.009 (2009). 19307040 10.1016/j.tree.2009.01.009

[40] Mutanen, M. et al. Species-level para-and polyphyly in DNA barcode gene trees: strong operational bias in European Lepidoptera. *Syst. Biol.* **65**, 1024–1040. 10.1093/sysbio/syw044 (2016). 27288478 10.1093/sysbio/syw044 PMC5066064

[41] Ballard, J. W. O. & Whitlock, M. C. The incomplete natural history of mitochondria. *Mol. Ecol.* **13**, 729–744. 10.1046/j.1365-294X.2003.02063.x (2004). 15012752 10.1046/j.1365-294x.2003.02063.x

[42] Tonzo, V., Papadopoulou, A. & Ortego, J. Genomic footprints of an old affair: single nucleotide polymorphism data reveal historical hybridization and the subsequent evolution of reproductive barriers in two recently diverged grasshoppers with partly overlapping distributions. *Mol. Ecol.* **29**, 2254–2268. 10.1111/mec.15475 (2020). 32418257 10.1111/mec.15475

[43] Benites, P. et al. Multiple introgression events during the diversification history of the edible Mexican grasshopper genus *Sphenarium* (Orthoptera: Pyrgomorphidae). *Mol. Phylogenet Evol.* **183**, 107774. 10.1016/j.ympev.2023.107774 (2023). 36972795 10.1016/j.ympev.2023.107774

[44] Kim, S. Y., Kang, T. H., Kim, T. W. & Seo, H. Y. DNA barcoding of the South Korean Tettigoniidae (Orthoptera) using collection specimens reveals three potential species complexes. *Entomol. Res.* **50**, 267–281. 10.1111/1748-5967.12433 (2020).

[45] Zhao, Y., Wang, H., Huang, H. & Zhou, Z. A DNA barcode library for katydids, cave crickets, and leaf-rolling crickets (Tettigoniidae, Rhaphidophoridae and Gryllacrididae) from Zhejiang Province. *China ZooKeys*. **1123**, 147–171. 10.3897/zookeys.1123.86704 (2022). 36762040 10.3897/zookeys.1123.86704 PMC9836636

[46] Zhou, Z. et al. Singleton molecular species delimitation based on COI-5P barcode sequences revealed high cryptic/undescribed diversity for Chinese Katydids (Orthoptera: Tettigoniidae). *BMC Evol. Biol.* **19**, 79. 10.1186/s12862-019-1404-5 (2019). 30871464 10.1186/s12862-019-1404-5 PMC6419471

[47] McCulloch, G. A., Dutoit, L., Craw, D., Kroos, G. C. & Waters, J. M. Genomics reveals exceptional phylogenetic diversity within a narrow-range flightless insect. *Insect Syst. Divers.* **6**, 5. 10.1093/isd/ixac009 (2022).

[48] Timm, V. F., Gonçalves, L. T., Valente, V. L. D. S. & Depra, M. The efficiency of the COI gene as a DNA barcode and an overview of Orthoptera (Caelifera and Ensifera) sequences in the BOLD system. *Can. J. Zool.* **100**, 710–718. 10.1139/cjz-2022-0041 (2022).

[49] Massa, B. & Fontana, P. Supraspecific taxonomy of palaearctic platycleidini with unarmed prosternum: a morphological approach (Orthoptera: tettigoniidae, Tettigoniinae). *Zootaxa* **2837**, 1–47. 10.11646/zootaxa.2837.1.1 (2011).

[50] Bland, L. M. et al. Toward reassessing data-deficient species. *Conserv. Biol.* **31**, 531–539. 10.1111/cobi.12850 (2017). 27696559 10.1111/cobi.12850

[51] Borgelt, J., Dorber, M., Høiberg, M. A. & Verones, F. More than half of data deficient species predicted to be threatened by extinction. *Commun. Biol.* **5**, 679. 10.1038/s42003-022-03638-9 (2022). 35927327 10.1038/s42003-022-03638-9 PMC9352662

[52] Barat, J. Revisión preliminar de Los Géneros de ephippigerini Brunner von wattenwyl, 1878 (Orthoptera: tettigoniidae: Bradyporinae). *Boletín De La. S E A*. **50**, 1–71 (2012).

[53] Harz, K. *Die Orthopteren Europas I. The Orthoptera of Europe, Series Entomologica* **5** (Dr. W. Junk N.V, (1969).

[54] Barat, J. Revisión taxonómica de Los ephippigerinae (Orthoptera: tettigonioidea: Bradyporidae) de La Península ibérica e Islas baleares, I. Géneros: Callicrania bolívar, 1898; Neocallicrania pfau, 1996; Platystolus bolívar, 1878 y synephippius navàs, 1905. *Boletín De La. S E A*. **40**, 55–118 (2007).

[55] Solé, J. et al. Range, population structure and morphological characterization of the small range endemic bush-cricket *Lluciapomaresius panteli* (Orthoptera: tettigoniidae: Bradyporinae). *J. Insect Conserv.* **22**, 659–674. 10.1007/s10841-018-0092-6 (2018).

[56] Domenech-fernández, M. & Llucià-Pomares, D. Contribución al Conocimiento taxonómico y Faunístico Del Género *Pycnogaster* graells, 1851: I. subgénero *Bradygaster* bolívar, 1926 (Orthoptera: tettigoniidae: Bradyporinae). *Matér Orthopt Entomocén*. **29**, 51–137 (2024).

[57] Folmer, O., Black, M., Hoeh, W., Lutz, R. & Vrijenhoek, R. DNA primers for amplification of mitochondrial cytochrome c oxidase subunit I from diverse metazoan invertebrates. *Mol. Mar. Biol. Biotechnol.* **3**, 294–299 (1994). 7881515

[58] Machordom, A., Araujo, R., Erpenbeck, D. & Ramos, M. Á. Phylogeography and conservation genetics of endangered European Margaritiferidae (Bivalvia: Unionoidea). *Biol. J. Linn. Soc.* **78**, 235–252. 10.1046/j.1095-8312.2003.00158.x (2003).

[59] Moulton, M. J., Song, H. & Whiting, M. F. Assessing the effects of primer specificity on eliminating Numt coamplification in DNA barcoding: a case study from Orthoptera (Arthropoda: Insecta). *Mol. Ecol. Resour.* **10**, 615–627. 10.1111/j.1755-0998.2009.02823.x (2010). 21565066 10.1111/j.1755-0998.2009.02823.x

[60] Minh, B. Q. et al. IQ-TREE 2: new models and efficient methods for phylogenetic inference in the genomic era. *Mol. Biol. Evol.* **37**, 1530–1534. 10.1093/molbev/msaa015 (2020). 32011700 10.1093/molbev/msaa015 PMC7182206

[61] Kalyaanamoorthy, S., Minh, B. Q., Wong, T. K., Von Haeseler, A. & Jermiin, L. S. ModelFinder: fast model selection for accurate phylogenetic estimates. *Nat. Methods*. **14**, 587–589. 10.1038/nmeth.4285 (2017). 28481363 10.1038/nmeth.4285 PMC5453245

[62] Hoang, D. T., Chernomor, O., Von Haeseler, A., Minh, B. Q. & Vinh, L. S. UFBoot2: improving the ultrafast bootstrap approximation. *Mol. Biol. Evol.* **35**, 518–522. 10.1093/molbev/msx281 (2018). 29077904 10.1093/molbev/msx281 PMC5850222

[63] Kimura, M. A simple method for estimating evolutionary rates of base substitutions through comparative studies of nucleotide sequences. *J. Mol. Evol.* **16**, 111–120. 10.1007/BF01731581 (1980). 7463489 10.1007/BF01731581

[64] Paradis, E., Claude, J. & Strimmer, K. APE: analyses of phylogenetics and evolution in R Language. *Bioinformatics* **20**, 289–290. 10.1093/bioinformatics/btg412 (2004). 14734327 10.1093/bioinformatics/btg412

