# Supplementary material for: DNA barcode reference library of bush-crickets (Orthoptera, Tettigoniidae) from the Iberian Peninsula
Source: Sci Rep. 2025 Sep 30;15:33883. doi: 10.1038/s41598-025-06695-2 (PMC12484681; doi:10.1038/s41598-025-06695-2)

Supplementary Information

**DNA barcode reference library of bush-crickets (Orthoptera, Tettigoniidae) from the Iberian Peninsula**

Jorge Gutiérrez-Rodríguez^1,2^, Alejandro Zaldívar-Riverón^3^, E. Karen López Estrada^4^, Pablo Barranco^5^, Mario García-París^2*^

1 School of Life and Environmental Sciences, University of Sydney, Sydney, NSW 2006, Australia

2 Department of Biodiversity and Evolutionary Biology. Museo Nacional de Ciencias Naturales (MNCN-CSIC), Madrid, Spain.

3 Colección Nacional de Insectos, Instituto de Biología, Universidad Nacional Autónoma de México, Ciudad de México, Mexico

4 Unidad de Síntesis en Sistemática y Evolución (UniSSE), Instituto de Biología, Universidad Nacional Autónoma de México, Ciudad de México, Mexico

5 Departamento de Biología y Geología, CITE-IIB, CECOUAL, Universidad de Almería, Ctra. Sacramento, s/n, 04120 La Cañada, Almería, Spain.

*corresponding author: Department of Biodiversity and Evolutionary Biology. Museo Nacional de Ciencias Naturales (MNCN-CSIC), Madrid, Spain. E-mail: [mparis@mncn.csic.es](mailto:mparis@mncn.csic.es)

Table S1. Locality information for the specimens examined in this study, including GenBank accession number, BOLD code, species assignment, mitochondrial introgression from a close species, coordinates, and locality. Sequences identified as IBIOR were obtained from Pina et al. (2024).

| **ID** | **Subfamily** | **Genbank** | **BOLD code** | **Species** | **Introgression Event** | **Latitude** | **Longitude** | **Country** | **Locality** |
| --- | --- | --- | --- | --- | --- | --- | --- | --- | --- |
| T091355 | Bradyporinae | PQ568702 | TETTI241-24 | *Albarracinia zapaterii* |  | 38.542 | -4.408 | Spain | Ciudad Real, Brazatortas, Puerto de Niefla |
| T091354 | Bradyporinae | PQ568701 | TETTI240-24 | *Albarracinia zapaterii* |  | 38.467 | -4.345 | Spain | Ciudad Real, Fuencaliente, Puerto de Valderrepisa |
| T090161 | Bradyporinae | PQ568522 | TETTI061-24 | *Baetica ustulata* |  | 37.101 | -3.043 | Spain | Granada, Aldeire, Collado del Realejo |
| T090162 | Bradyporinae | PQ568523 | TETTI062-24 | *Baetica ustulata* |  | 37.101 | -3.043 | Spain | Granada, Aldeire, Collado del Realejo |
| T092735 | Bradyporinae | PQ568807 | TETTI347-24 | *Baratia sari* |  | 42.546 | 1.708 | Andorra | Encamp |
| T092736 | Bradyporinae | PQ568808 | TETTI348-24 | *Baratia sari* |  | 42.546 | 1.708 | Andorra | Encamp |
| T091509 | Bradyporinae | PQ568726 | TETTI265-24 | *Callicrania demandae* |  | 42.224 | -2.977 | Spain | La Rioja, Ezcaray, Valdezcaray |
| T091510 | Bradyporinae | PQ568727 | TETTI266-24 | *Callicrania demandae* |  | 42.224 | -2.977 | Spain | La Rioja, Ezcaray, Valdezcaray |
| T090607 | Bradyporinae | PQ568588 | TETTI127-24 | *Callicrania faberi* |  | 42.902 | -5.782 | Spain | León, La Pola de Gordón |
| T091553 | Bradyporinae | PQ568731 | TETTI270-24 | *Callicrania plaxicauda* |  | 42.371 | 1.237 | Spain | Lleida, Soriguera |
| T091554 | Bradyporinae | PQ568732 | TETTI271-24 | *Callicrania plaxicauda* |  | 42.371 | 1.237 | Spain | Lleida, Soriguera |
| T090357 | Bradyporinae | PQ568548 | TETTI087-24 | *Callicrania ramburii* |  | 43.275 | -4.833 | Spain | Asturias, Cabrales, Bulnes |
| T092063 | Bradyporinae | PQ568777 | TETTI316-24 | *Callicrania vicentae* |  | 41.185 | -3.473 | Spain | Segovia, Cerezo de Arriba |
| MNCN206965 | Bradyporinae | PQ568504 | TETTI043-24 | *Coracinotus notaroi notaroi* |  | 38.527 | -2.425 | Spain | Albacete, Riopar, Sierra de Alcaraz |
| MNCN206966 | Bradyporinae | PQ568505 | TETTI044-24 | *Coracinotus notaroi notaroi* |  | 38.527 | -2.425 | Spain | Albacete, Riopar, Sierra de Alcaraz |
| T092170 | Bradyporinae | PQ568790 | TETTI329-24 | *Coracinotus notaroi lluciapomaresi* |  | 38.319 | -2.576 | Spain | Jaén, Siles, Las Acebedas |
| T092169 | Bradyporinae | PQ568789 | TETTI328-24 | *Coracinotus politus* |  | 38.057 | -2.552 | Spain | Granada, Huecar, La Vidriera |
| T091504 | Bradyporinae | PQ568725 | TETTI264-24 | *Ephippiger diurnus* |  | 42.701 | -3.276 | Spain | Burgos, Oña, La Aldea |
| T090687 | Bradyporinae | PQ568606 | TETTI145-24 | *Ephippiger diurnus* |  | 42.773 | 0.681 | Spain | Lleida, Bossòst, Puerto del Portillón |
| T090726 | Bradyporinae | PQ568614 | TETTI153-24 | *Ephippiger diurnus* |  | 42.773 | 0.681 | Spain | Lleida, Bossòst, Puerto del Portillón |
| T090744 | Bradyporinae | PQ568617 | TETTI156-24 | *Ephippigerida areolaria* |  | 40.5 | -1.487 | Spain | Teruel, Monterde de Albarracín |
| T091312 | Bradyporinae | PQ568690 | TETTI229-24 | *Ephippigerida* aff. *areolaria* |  | 40.979 | -3.847 | Spain | Madrid, Pinilla del Valle, Pico del Nevero |
| T092047 | Bradyporinae | PQ568774 | TETTI313-24 | *Ephippigerida asella* |  | 42.297 | 1.174 | Spain | Lleida, Les Valls d'Aguilar, Els Castells |
| T092048 | Bradyporinae | PQ568775 | TETTI314-24 | *Ephippigerida asella* |  | 42.297 | 1.174 | Spain | Lleida, Les Valls d'Aguilar, Els Castells |
| T092172 | Bradyporinae | PQ568791 | TETTI330-24 | *Ephippigerida barati* |  | 38.919 | -2.599 | Spain | Albacete, El Bonillo |
| TTT159 | Bradyporinae | PQ568826 | TETTI366-24 | *Ephippigerida carinata* |  | 39.878 | -2.794 | Spain | Cuenca, 5 km S Saelices |
| T091447 | Bradyporinae | PQ568716 | TETTI255-24 | *Ephippigerida carinata* |  | 40.073 | -2.312 | Spain | Cuenca, Fuentenava de Jábaga, Puerto de Cabrejas |
| TTT168 | Bradyporinae | PQ568829 | TETTI369-24 | *Ephippigerida carinata* |  | 40.146 | -3.125 | Spain | Madrid, 6 km SO Estremera |
| T090499 | Bradyporinae | PQ568574 | TETTI113-24 | *Ephippigerida diluta* |  | 41.432 | -7.002 | Portugal | Bragança, Alfândega da Fé, Sambade, Serra de Bornes |
| **ID** | **Subfamily** | **Genbank** | **BOLD code** | **Species** | **Introgression Event** | **Latitude** | **Longitude** | **Country** | **Locality** |
| T091258 | Bradyporinae | PQ568684 | TETTI223-24 | *Ephippigerida diluta* |  | 41.233 | -3.213 | Spain | Guadalajara, Cantalojas |
| T090728 | Bradyporinae | PQ568615 | TETTI154-24 | *Ephippigerida diluta* |  | 42.016 | -6.347 | Spain | Zamora, Mombuey |
| T090688 | Bradyporinae | PQ568607 | TETTI146-24 | *Ephippigerida* aff. *diluta* 1 |  | 41.246 | -7.884 | Portugal | Porto, Teixeiró, Antenas do Marão |
| INV08107 | Bradyporinae | OR974778 | IBIOR258-22 | *Ephippigerida* aff*. diluta* 2 |  | 40.301 | -7.095 | Portugal | Castelo Branco, Penamacor, Meimao |
| INV08108 | Bradyporinae | OR974678 | IBIOR259-22 | *Ephippigerida* aff*. diluta* 2 |  | 40.301 | -7.095 | Portugal | Castelo Branco, Penamacor, Meimao |
| T092292 | Bradyporinae | PQ568804 | TETTI344-24 | *Ephippigerida fernandezi* |  | 39.562 | -5.983 | Spain | Cáceres, Trujillo |
| T091608 | Bradyporinae | PQ568735 | TETTI274-24 | *Ephippigerida laserena* |  | 38.77 | -5.515 | Spain | Badajoz, Castuera |
| T091609 | Bradyporinae | PQ568736 | TETTI275-24 | *Ephippigerida laserena* |  | 38.77 | -5.515 | Spain | Badajoz, Castuera |
| T091944 | Bradyporinae | PQ568763 | TETTI302-24 | *Ephippigerida pantingana* |  | 41.714 | -0.688 | Spain | Zaragoza, Villamayor de Gallego |
| INV08109 | Bradyporinae | OR974415 | IBIOR260-22 | *Ephippigerida rosae* |  | 39.173 | -8.965 | Portugal | Lisboa, Azambuja, Alcoentre |
| T091668 | Bradyporinae | PQ568747 | TETTI286-24 | *Ephippigerida rosae* |  | 39.174 | -9.085 | Portugal | Lisboa, Vila Verde dos Francos |
| T091669 | Bradyporinae | PQ568748 | TETTI287-24 | *Ephippigerida rosae* |  | 39.174 | -9.085 | Portugal | Lisboa, Vila Verde dos Francos |
| TTT204 | Bradyporinae | PQ568836 | TETTI376-24 | *Ephippigerida saussuriana* |  | 42.714 | -4.12 | Spain | Burgos, Quintanas de Valdelucio |
| T091271 | Bradyporinae | PQ568687 | TETTI226-24 | *Lluciapomaresius anapaulae* |  | 39.418 | -8.92 | Portugal | Leiria, Chões |
| T091273 | Bradyporinae | PQ568688 | TETTI227-24 | *Lluciapomaresius anapaulae* |  | 39.515 | -8.78 | Portugal | Leiria, Covão do Sabugueiro |
| T091274 | Bradyporinae | PQ568689 | TETTI228-24 | *Lluciapomaresius anapaulae* |  | 39.515 | -8.78 | Portugal | Leiria, Covão do Sabugueiro |
| INV02573 | Bradyporinae | OR974723 | IBIOR129-17 | *Lluciapomaresius anapaulae* |  | 39.502 | -8.828 | Portugal | Leiria, Porto de Mos, Mendiga |
| INV05560 | Bradyporinae | OR974648 | IBIOR211-22 | *Lluciapomaresius asturiensis* |  | 41.835 | -7.946 | Portugal | Vila Real, Montalegre, Pitoes das Junias |
| INV05561 | Bradyporinae | OR974697 | IBIOR212-22 | *Lluciapomaresius asturiensis* |  | 41.835 | -7.946 | Portugal | Vila Real, Montalegre, Pitoes das Junias |
| T091474 | Bradyporinae | PQ568722 | TETTI261-24 | *Lluciapomaresius asturiensis* |  | 43.057 | -6.09 | Spain | León, San Emiliano, Alto de la Farrapona |
| T090710 | Bradyporinae | PQ568610 | TETTI149-24 | *Lluciapomaresius asturiensis* |  | 43.008 | -5.683 | Spain | León, Tonín de Arbas |
| T091396 | Bradyporinae | PQ568707 | TETTI246-24 | *Lluciapomaresius asturiensis* |  | 42.631 | -7.893 | Spain | Pontevedra, Rodeiro, Monte do Faro |
| T091230 | Bradyporinae | PQ568682 | TETTI221-24 | *Lluciapomaresius eclipticus* |  | 41.527 | -1.734 | Spain | Zaragoza, Villaroya de la Sierra, Sierra de la Virgen |
| T092088 | Bradyporinae | PQ568783 | TETTI322-24 | *Lluciapomaresius nisae* (=*L. panteli*) |  | 41.054 | 0.792 | Spain | Tarragona, Mola Perelló, Tivissa |
| T092089 | Bradyporinae | PQ568784 | TETTI323-24 | *Lluciapomaresius nisae* (=*L. panteli*) |  | 41.054 | 0.792 | Spain | Tarragona, Mola Perelló, Tivissa |
| INV08100 | Bradyporinae | OR974783 | IBIOR251-22 | *Lluciapomaresius nobrei* | # *L. stalii* | 40.350 | -7.549 | Portugal | Guarda, Manteigas, Parque Natural da Serra da Estrela |
| INV03952 | Bradyporinae | OR974672 | IBIOR333-22 | *Lluciapomaresius nobrei* |  | 40.365 | -7.636 | Portugal | Guarda, Seia, Lagoa Comprida |
| T090534 | Bradyporinae | PQ568584 | TETTI123-24 | *Lluciapomaresius nobrei* |  | 40.315 | -7.578 | Portugal | Guarda, Seia, Loriga, Serra da Estrela |
| T091532 | Bradyporinae | PQ568728 | TETTI267-24 | *Lluciapomaresius nobrei* |  | 40.338 | -7.616 | Portugal | Guarda, Seia, Loriga, Serra da Estrela |
| INV03388 | Bradyporinae | OR974819 | IBIOR307-22 | *Lluciapomaresius nobrei* |  | 40.385 | -7.618 | Portugal | Guarda, Seia, Near Vale Rossim |
| T091218 | Bradyporinae | PQ568680 | TETTI219-24 | *Lluciapomaresius ortegai* |  | 40.671 | -2.135 | Spain | Guadalajara, Peñalén |
| T091219 | Bradyporinae | PQ568681 | TETTI220-24 | *Lluciapomaresius ortegai* |  | 40.671 | -2.135 | Spain | Guadalajara, Peñalén |
| T092091 | Bradyporinae | PQ568785 | TETTI324-24 | *Lluciapomaresius panteli* |  | 41.3 | 0.911 | Spain | Tarragona, Cornudella de Montsant, Albarca, Sierra de Montsant |
| T092092 | Bradyporinae | PQ568786 | TETTI325-24 | *Lluciapomaresius panteli* |  | 41.3 | 0.911 | Spain | Tarragona, Cornudella de Montsant, Albarca, Sierra de Montsant |
| **ID** | **Subfamily** | **Genbank** | **BOLD code** | **Species** | **Introgression Event** | **Latitude** | **Longitude** | **Country** | **Locality** |
| T091966 | Bradyporinae | PQ568766 | TETTI305-24 | *Lluciapomaresius panteli* |  | 41.292 | 0.894 | Spain | Tarragona, La Morera de Montsant |
| T091244 | Bradyporinae | PQ568683 | TETTI222-24 | *Lluciapomaresius stalii* |  | 41.233 | -3.213 | Spain | Guadalajara, Cantalojas |
| T090716 | Bradyporinae | PQ568611 | TETTI150-24 | *Lluciapomaresius stalii* |  | 40.604 | -4.174 | Spain | Madrid, San Lorenzo de El Escorial, Puerto de Malagón |
| T090704 | Bradyporinae | PQ568609 | TETTI148-24 | *Lluciapomaresius stalii* |  | 41.134 | -3.587 | Spain | Madrid, Somosierra |
| INV08101 | Bradyporinae | OR974485 | IBIOR252-22 | *Neocallicrania barrosi* |  | 39.730 | -9.034 | Portugal | Leiria, Alcobaca, Pataias |
| T091664 | Bradyporinae | PQ568745 | TETTI284-24 | *Neocallicrania barrosi* |  | 39.393 | -9.262 | Portugal | Leiria, Amoreira |
| T091665 | Bradyporinae | PQ568746 | TETTI285-24 | *Neocallicrania barrosi* |  | 39.393 | -9.262 | Portugal | Leiria, Amoreira |
| INV08102 | Bradyporinae | OR974563 | IBIOR253-22 | *Neocallicrania barrosi* |  | 39.624 | -9.074 | Portugal | Leiria, Nazare, Nazare |
| T091381 | Bradyporinae | PQ568705 | TETTI244-24 | *Neocallicrania bolivarii* |  | 43.362 | -6.973 | Spain | Asturias, Villanueva de Oscos, Sierra de la Bobia |
| T090459 | Bradyporinae | PQ568569 | TETTI108-24 | *Neocallicrania bolivarii* |  | 42.634 | -9.038 | Spain | Galicia, Grandemaior |
| T091388 | Bradyporinae | PQ568706 | TETTI245-24 | *Neocallicrania bolivarii* |  | 42.63 | -7.893 | Spain | Pontevedra, Rodeiro, Monte do Faro |
| INV08110 | Bradyporinae | OR974578 | IBIOR261-22 | *Neocallicrania lusitanica* |  | 39.165 | -8.959 | Portugal | Lisboa, Azambuja, Alcoentre |
| INV08111 | Bradyporinae | OR974435 | IBIOR262-22 | *Neocallicrania lusitanica* |  | 39.165 | -8.959 | Portugal | Lisboa, Azambuja, Alcoentre |
| T090947 | Bradyporinae | PQ568655 | TETTI194-24 | *Neocallicrania lusitanica* |  | 39.313 | -7.36 | Portugal | Portalegre, Reguengo |
| T091695 | Bradyporinae | PQ568756 | TETTI295-24 | *Neocallicrania lusitanica* |  | 39.308 | -7.36 | Portugal | Portalegre, Reguengo |
| INV08112 | Bradyporinae | OR974827 | IBIOR263-22 | *Neocallicrania lusitanica* |  | 38.458 | -9.025 | Portugal | Setubal, Setubal, Parque Natural da Arrabida, Vale do Risco |
| T091424 | Bradyporinae | PQ568711 | TETTI250-24 | *Neocallicrania lusitanica* |  | 39.214 | -6.127 | Spain | Cáceres, Montánchez |
| T092065 | Bradyporinae | PQ568779 | TETTI318-24 | *Neocallicrania lusitanica* |  | 37.077 | -6.689 | Spain | Huelva, Almonte, Cuesta Maneli |
| INV08103 | Bradyporinae | OR974490 | IBIOR254-22 | *Neocallicrania miegii* | # *N. lusitanica* | 40.350 | -7.549 | Portugal | Guarda, Manteigas, Parque Natural da Serra da Estrela |
| INV08104 | Bradyporinae | OR974513 | IBIOR255-22 | *Neocallicrania miegii* | # *N. lusitanica* | 40.350 | -7.549 | Portugal | Guarda, Manteigas, Parque Natural da Serra da Estrela |
| T090731 | Bradyporinae | PQ568616 | TETTI155-24 | *Neocallicrania miegii* |  | 40.273 | -5.662 | Spain | Cáceres, Tornavacas, Puerto de Tornavacas |
| T090745 | Bradyporinae | PQ568618 | TETTI157-24 | *Neocallicrania miegii* |  | 40.587 | -1.613 | Spain | Guadalajara, Motos |
| T090747 | Bradyporinae | PQ568619 | TETTI158-24 | *Neocallicrania miegii* |  | 40.59 | -4.193 | Spain | Madrid, Robledondo |
| T091182 | Bradyporinae | PQ568677 | TETTI216-24 | *Neocallicrania selligera selligera* |  | 41.768 | -8.216 | Portugal | Braga, Terras de Bouro, Serra do Gerês |
| INV06585 | Bradyporinae | OR974724 | IBIOR429-22 | *Neocallicrania selligera selligera* |  | 41.315 | -7.220 | Portugal | Bragança, Vila Flor, Freixiel |
| T091471 | Bradyporinae | PQ568721 | TETTI260-24 | *Neocallicrania selligera selligera* |  | 42.195 | -3.305 | Spain | Burgos, Pineda de la Sierra, Estación Invernal Valle del Sol |
| T090472 | Bradyporinae | PQ568571 | TETTI110-24 | *Neocallicrania selligera selligera* |  | 42.04 | -7.859 | Spain | Ourense, Xinzo de Lima, Congostro |
| INV04091 | Bradyporinae | OR974528 | IBIOR354-22 | *Neocallicrania selligera meridionalis* |  | 40.325 | -7.572 | Portugal | Guarda, Gouveia, S. Pedro (birches) |
| T091158 | Bradyporinae | PQ568675 | TETTI214-24 | *Neocallicrania selligera meridionalis* |  | 40.372 | -7.516 | Portugal | Guarda, Manteigas, Serra Estrela |
| INV07436 | Bradyporinae | OR974462 | IBIOR470-22 | *Neocallicrania selligera meridionalis* |  | 40.385 | -7.705 | Portugal | Guarda, Seia, Hydroelectric power plant of Ponte de Jugais |
| T091673 | Bradyporinae | PQ568749 | TETTI288-24 | *Neocallicrania selligera meridionalis* |  | 39.182 | -9.058 | Portugal | Lisboa, Lamas |
| INV02574 | Bradyporinae | OR974631 | IBIOR130-17 | *Neocallicrania selligera meridionalis* |  | 39.502 | -8.828 | Portugal | Porto de Mos, Mendiga |
| MNCN101183 | Bradyporinae | PQ568503 | TETTI042-24 | *Neocallicrania serrata serrata* |  | 38.091 | -8.795 | Portugal | Setúbal, Costa de Santo André, Vila Nova de Santo André |
| INV08105 | Bradyporinae | OR974605 | IBIOR256-22 | *Neocallicrania serrata serrata* |  | 38.126 | -8.789 | Portugal | Setubal, Grandola, Melides |
| **ID** | **Subfamily** | **Genbank** | **BOLD code** | **Species** | **Introgression Event** | **Latitude** | **Longitude** | **Country** | **Locality** |
| INV08106 | Bradyporinae | OR974589 | IBIOR257-22 | *Neocallicrania serrata serrata* |  | 38.126 | -8.789 | Portugal | Setubal, Grandola, Melides |
| T092198 | Bradyporinae | PQ568794 | TETTI334-24 | *Neocallicrania serrata pfaui* |  | 37.306 | -8.812 | Portugal | Faro, Aljezur |
| T092234 | Bradyporinae | PQ568798 | TETTI338-24 | *Neocallicrania serrata pfaui* |  | 37.028 | -8.971 | Portugal | Cabo de San Vicente, Praia do Beliche |
| T091101 | Bradyporinae | PQ568672 | TETTI211-24 | *Parasteropleurus perezii* |  | 38.657 | -0.24 | Spain | Alicante, Benifato, Sierra de Aitana |
| T090319 | Bradyporinae | PQ568540 | TETTI079-24 | *Parasteropleurus perezii* |  | 40.193 | -1.443 | Spain | Teruel, Jabaloyas |
| INV02526 | Bradyporinae | OR974818 | IBIOR091-17 | *Platystolus martinezii* |  | 37.868 | -8.121 | Portugal | Beja, Aljustrel, Aljustrel |
| INV02527 | Bradyporinae | OR974545 | IBIOR092-17 | *Platystolus martinezii* |  | 37.868 | -8.123 | Portugal | Beja, Aljustrel, Aljustrel |
| T091593 | Bradyporinae | PQ568733 | TETTI272-24 | *Platystolus martinezii* |  | 38.77 | -5.515 | Spain | Badajoz, Castuera |
| T090694 | Bradyporinae | PQ568608 | TETTI147-24 | *Platystolus martinezii* |  | 42.718 | -4.127 | Spain | Burgos, Quintanas de Valdelucio |
| T091432 | Bradyporinae | PQ568714 | TETTI253-24 | *Platystolus surcularius* |  | 39.462 | -3.176 | Spain | Ciudad Real, Campo de Criptana, Laguna de Salicor |
| T091430 | Bradyporinae | PQ568713 | TETTI252-24 | *Platystolus surcularius* |  | 39.859 | -2.675 | Spain | Cuenca, Montalbo, El Hito |
| INV05577 | Bradyporinae | OR974622 | IBIOR228-22 | *Pycnogaster cucullatus* | # *P. jugicola* | 39.015 | -8.448 | Portugal | Santarem, Coruche, Herdade dos Concelhos |
| T091075 | Bradyporinae | PQ568669 | TETTI208-24 | *Pycnogaster cucullatus* |  | 39.432 | -8.923 | Portugal | Santarém, Rio Maior, Alcobertas, Serra dos Candeeiros |
| INV02590 | Bradyporinae | OR974521 | IBIOR180-22 | *Pycnogaster cucullatus* |  | 38.417 | -9.193 | Portugal | Setubal, Sesimbra, Cabo Espichel |
| T091635 | Bradyporinae | PQ568741 | TETTI280-24 | *Pycnogaster gaditana* |  | 36.127 | -5.706 | Spain | Cádiz, Tarifa, Puerto de Facinas |
| T091343 | Bradyporinae | PQ568695 | TETTI234-24 | *Pycnogaster graellsii* |  | 39.863 | -2.705 | Spain | Cuenca, Montalbo, Laguna de El Hito |
| T091349 | Bradyporinae | PQ568697 | TETTI236-24 | *Pycnogaster graellsii* |  | 39.863 | -2.705 | Spain | Cuenca, Montalbo, Laguna de El Hito |
| T090834 | Bradyporinae | PQ568635 | TETTI174-24 | *Pycnogaster graellsii* |  | 39.514 | -3.344 | Spain | Toledo, Villacañas, Laguna de Peñahueca |
| T090821 | Bradyporinae | PQ568623 | TETTI162-24 | *Pycnogaster inermis* |  | 37.264 | -2.778 | Spain | Almería, Baza, Puerto de Escullar |
| T090822 | Bradyporinae | PQ568624 | TETTI163-24 | *Pycnogaster inermis* |  | 37.201 | -2.606 | Spain | Almería, Gérgal, 6 km W Calar Alto |
| T090835 | Bradyporinae | PQ568636 | TETTI175-24 | *Pycnogaster inermis* |  | 37.213 | -2.672 | Spain | Almería, Nacimiento, Sierra de Filabres |
| T090823 | Bradyporinae | PQ568625 | TETTI164-24 | *Pycnogaster inermis* |  | 37.096 | -3.385 | Spain | Granada, Güejar Sierra, Sierra Nevada, Peñones de San Francisco |
| T090838 | Bradyporinae | PQ568639 | TETTI178-24 | *Pycnogaster inermis* |  | 37.1 | -2.977 | Spain | Granada, Huéneja, Sierra Nevada, El Anillo |
| T090839 | Bradyporinae | PQ568640 | TETTI179-24 | *Pycnogaster inermis* |  | 37.63 | -3.775 | Spain | Jaen, Valdepeñas de Jaén, Alto de la Pandera |
| T090840 | Bradyporinae | PQ568641 | TETTI180-24 | *Pycnogaster inermis* |  | 37.63 | -3.775 | Spain | Jaen, Valdepeñas de Jaén, Alto de la Pandera |
| T091353 | Bradyporinae | PQ568700 | TETTI239-24 | *Pycnogaster inermis* |  | - | - | Spain | Jaén, Valdepeñas de Jaén, Sierra de la Pandera |
| T091150 | Bradyporinae | PQ568674 | TETTI213-24 | *Pycnogaster jugicola* |  | 40.86 | -8.26 | Portugal | Aveiro, Albergaria da Serra, Serra da Freita |
| INV05563 | Bradyporinae | OR974704 | IBIOR214-22 | *Pycnogaster jugicola* |  | 41.863 | -7.953 | Portugal | Vila Real, Montalegre, Pitoes das Junias, near Mosteiro |
| TTT163 | Bradyporinae | PQ568827 | TETTI367-24 | *Pycnogaster jugicola* |  | 40.652 | -4.209 | Spain | Ávila, Peguerinos, Camping de Peguerinos |
| T090831 | Bradyporinae | PQ568633 | TETTI172-24 | *Pycnogaster jugicola* |  | 40.621 | -4.179 | Spain | Ávila, Peguerinos, Puerto de Malagón |
| T090828 | Bradyporinae | PQ568630 | TETTI169-24 | *Pycnogaster jugicola* |  | 40.587 | -1.613 | Spain | Guadalajara, Motos |
| T090829 | Bradyporinae | PQ568631 | TETTI170-24 | *Pycnogaster jugicola* |  | 40.587 | -1.613 | Spain | Guadalajara, Motos |
| T090825 | Bradyporinae | PQ568627 | TETTI166-24 | *Pycnogaster jugicola* |  | 40.602 | -4.165 | Spain | Madrid, El Escorial |
| T090826 | Bradyporinae | PQ568628 | TETTI167-24 | *Pycnogaster jugicola* |  | 40.602 | -4.165 | Spain | Madrid, El Escorial |
| **ID** | **Subfamily** | **Genbank** | **BOLD code** | **Species** | **Introgression Event** | **Latitude** | **Longitude** | **Country** | **Locality** |
| TTT182 | Bradyporinae | PQ568832 | TETTI372-24 | *Pycnogaster jugicola* |  | 40.707 | -4.141 | Spain | Madrid, Guadarrama, Puerto Guadarrama |
| T090820 | Bradyporinae | PQ568622 | TETTI161-24 | *Pycnogaster jugicola* |  | 40.835 | -3.839 | Spain | Madrid, Rascafría, Puerto de la Morcuera |
| T090836 | Bradyporinae | PQ568637 | TETTI176-24 | *Pycnogaster jugicola* |  | 40.336 | -5.731 | Spain | Salamanca, Candelario, Sierra de Bejar, El Travieso |
| T090837 | Bradyporinae | PQ568638 | TETTI177-24 | *Pycnogaster jugicola* |  | 40.336 | -5.731 | Spain | Salamanca, Candelario, Sierra de Bejar, El Travieso |
| TTT158 | Bradyporinae | PQ568825 | TETTI365-24 | *Pycnogaster jugicola* |  | 40.818 | -3.964 | Spain | Segovia, San Ildefonso, Puerto de Cotos |
| T090819 | Bradyporinae | PQ568621 | TETTI160-24 | *Pycnogaster jugicola* |  | 40.501 | -1.589 | Spain | Teruel, Bronchales |
| T090827 | Bradyporinae | PQ568629 | TETTI168-24 | *Pycnogaster jugicola* |  | 40.369 | -1.739 | Spain | Teruel, Guadalaviar, Puerto del Portillo |
| T090830 | Bradyporinae | PQ568632 | TETTI171-24 | *Pycnogaster jugicola* |  | 40.369 | -1.739 | Spain | Teruel, Guadalaviar, Puerto del Portillo |
| T090824 | Bradyporinae | PQ568626 | TETTI165-24 | *Pycnogaster jugicola* |  | 40.558 | -1.483 | Spain | Teruel, Pozondón |
| T090833 | Bradyporinae | PQ568634 | TETTI173-24 | *Pycnogaster* rosae |  | 36.845 | -2.559 | Spain | Almería, Enix, Sierra de Gador, Lomas de las Minas |
| T091110 | Bradyporinae | PQ568673 | TETTI212-24 | *Pycnogaster sanchezgomezi* |  | 38.65 | -0.225 | Spain | Alicante, Benifato, Puerto de Tagarina |
| T091350 | Bradyporinae | PQ568698 | TETTI237-24 | *Pycnogaster sanchezgomezi* |  | 38.042 | -2.472 | Spain | Granada, Puebla de Don Fadrique, Sierra de Guillimona |
| T092291 | Bradyporinae | PQ568803 | TETTI343-24 | *Pycnogaster ribesiglesiasii* (=*P. sanchezgomezi*) |  | 41.459 | 1.507 | Spain | Barcelona, La Llacuna, Altiplano d'Ancosa |
| T092288 | Bradyporinae | PQ568800 | TETTI340-24 | *Pycnogaster* sp. nov. 1 - Algarve |  | 37.159 | -7.909 | Portugal | Faro, São Brás de Alportel |
| T092289 | Bradyporinae | PQ568801 | TETTI341-24 | *Pycnogaster* sp. nov. 1 - Algarve |  | 37.159 | -7.909 | Portugal | Faro, São Brás de Alportel |
| T090857 | Bradyporinae | PQ568644 | TETTI183-24 | *Pycnogaster valentini* |  | 39.465 | -5.35 | Spain | Cáceres, Guadalupe, Ermita del Humilladero |
| T090858 | Bradyporinae | PQ568645 | TETTI184-24 | *Pycnogaster valentini* |  | 39.465 | -5.35 | Spain | Cáceres, Guadalupe, Ermita del Humilladero |
| T090859 | Bradyporinae | PQ568646 | TETTI185-24 | *Pycnogaster valentini* |  | 39.465 | -5.35 | Spain | Cáceres, Guadalupe, Ermita del Humilladero |
| T090860 | Bradyporinae | PQ568647 | TETTI186-24 | *Pycnogaster valentini* |  | 39.465 | -5.35 | Spain | Cáceres, Guadalupe, Ermita del Humilladero |
| T091352 | Bradyporinae | PQ568699 | TETTI238-24 | *Pycnogaster valentini* |  | - | - | Spain | Cáceres, Navalvillar del Pedroso |
| T091334 | Bradyporinae | PQ568691 | TETTI230-24 | *Pycnogaster valentini* |  | 38.542 | -4.408 | Spain | Ciudad Real, Brazatortas, Puerto de Niefla |
| T091335 | Bradyporinae | PQ568692 | TETTI231-24 | *Pycnogaster valentini* |  | 38.542 | -4.408 | Spain | Ciudad Real, Brazatortas, Puerto de Niefla |
| T091336 | Bradyporinae | PQ568693 | TETTI232-24 | *Pycnogaster valentini* |  | 38.542 | -4.408 | Spain | Ciudad Real, Brazatortas, Puerto de Niefla |
| T091342 | Bradyporinae | PQ568694 | TETTI233-24 | *Pycnogaster valentini* |  | 39.524 | -4.358 | Spain | Toledo, San Pablo de los Montes, Puerto del Robledillo |
| T091940 | Bradyporinae | PQ568762 | TETTI301-24 | *Sabaterpia taeniata* |  | 36.149 | -5.707 | Spain | Cádiz, Facinas |
| T092002 | Bradyporinae | PQ568768 | TETTI307-24 | *Sorapagus catalaunicus* |  | 42.162 | 1.403 | Spain | Lleida, Fígols i Alinyà |
| T092005 | Bradyporinae | PQ568769 | TETTI308-24 | *Sorapagus catalaunicus* |  | 42.107 | 1.389 | Spain | Lleida, Lladurs |
| T091550 | Bradyporinae | PQ568729 | TETTI268-24 | *Sorapagus catalaunicus* |  | 42.371 | 1.237 | Spain | Lleida, Soriguera |
| T091551 | Bradyporinae | PQ568730 | TETTI269-24 | *Sorapagus catalaunicus* |  | 42.371 | 1.237 | Spain | Lleida, Soriguera |
| INV02592 | Bradyporinae | OR974515 | IBIOR182-22 | *Steropleurus* aff*. pseudolus* |  | 38.423 | -9.197 | Portugal | Setubal, Sesimbra, Cabo Espichel |
| INV02593 | Bradyporinae | OR974620 | IBIOR183-22 | *Steropleurus* aff*. pseudolus* |  | 38.423 | -9.197 | Portugal | Setubal, Sesimbra, Cabo Espichel |
| T090723 | Bradyporinae | PQ568613 | TETTI152-24 | *Steropleurus andalusicus* |  | 36.999 | -2.891 | Spain | Almería, Láujar de Andarax |
| T092120 | Bradyporinae | PQ568788 | TETTI327-24 | *Steropleurus andalusicus* |  | 37.686 | -2.191 | Spain | Almería, María, Sierra Maria |
| T090165 | Bradyporinae | PQ568524 | TETTI063-24 | *Steropleurus andalusicus* |  | 36.927 | -3.288 | Spain | Granada, 3 km al N de Almegíjar |
| **ID** | **Subfamily** | **Genbank** | **BOLD code** | **Species** | **Introgression Event** | **Latitude** | **Longitude** | **Country** | **Locality** |
| T091462 | Bradyporinae | PQ568718 | TETTI257-24 | *Steropleurus andalusicus* |  | 37.133 | -3.434 | Spain | Granada, Güejar Sierra, Sierra Nevada |
| T091452 | Bradyporinae | PQ568717 | TETTI256-24 | *Steropleurus brunnerii* |  | 39.415 | -3.287 | Spain | Ciudad Real, Alcázar de San Juan, Laguna de las Yegüas |
| T091463 | Bradyporinae | PQ568719 | TETTI258-24 | *Steropleurus brunnerii* |  | 38.325 | -3.583 | Spain | Jaén, Navas de Tolosa, La Aligada |
| T091464 | Bradyporinae | PQ568720 | TETTI259-24 | *Steropleurus brunnerii* |  | 38.325 | -3.583 | Spain | Jaén, Navas de Tolosa, La Aligada |
| T091440 | Bradyporinae | PQ568715 | TETTI254-24 | *Steropleurus brunnerii* |  | 40.672 | -3.584 | Spain | Madrid, El Molar |
| T091614 | Bradyporinae | PQ568737 | TETTI276-24 | *Steropleurus* cf. *flavovittatus* |  | 38.77 | -5.515 | Spain | Badajoz, Castuera |
| T090169 | Bradyporinae | PQ568528 | TETTI067-24 | *Steropleurus* cf. *flavovittatus* |  | 37.679 | -3.534 | Spain | Jaén, 4 km al E de Cambil |
| T092116 | Bradyporinae | PQ568787 | TETTI326-24 | *Steropleurus* cf. *flavovittatus* |  | 37.956 | -5.619 | Spain | Sevilla, San Nicolás del Puerto, Cerro de Hierro |
| T091650 | Bradyporinae | PQ568744 | TETTI283-24 | *Steropleurus pseudolus* |  | 38.054 | -6.329 | Spain | Badajoz, Calera de León |
| T091957 | Bradyporinae | PQ568764 | TETTI303-24 | *Steropleurus pseudolus* |  | 37.869 | -6.752 | Spain | Huelva, Almonaster la Real |
| T092070 | Bradyporinae | PQ568780 | TETTI319-24 | *Steropleurus recticarinatus* |  | 37.077 | -6.689 | Spain | Huelva, Almonte, Cuesta Maneli |
| T092072 | Bradyporinae | PQ568781 | TETTI320-24 | *Steropleurus recticarinatus* |  | 37.077 | -6.689 | Spain | Huelva, Almonte, Cuesta Maneli |
| T090617 | Bradyporinae | PQ568589 | TETTI128-24 | *Steropleurus* sp. nov. 1 - Sª Nevada |  | 37.085 | -3.375 | Spain | Granada, Güejar Sierra, Sierra Nevada, Albergue universitario |
| T092233 | Bradyporinae | PQ568797 | TETTI337-24 | *Steropleurus* sp. nov. 2 - Algarve |  | 37.316 | -8.594 | Portugal | Faro, Monchique |
| T091362 | Bradyporinae | PQ568703 | TETTI242-24 | *Steropleurus* sp. nov. 2 - Algarve |  | 37.805 | -8.689 | Portugal | Setúbal, Cercal do Alentejo |
| T090722 | Bradyporinae | PQ568612 | TETTI151-24 | *Synephippius obvius* |  | 42.375 | -0.805 | Spain | Huesca, Agüero, Puerto de Sierra Alta |
| T090083 | Bradyporinae | PQ568512 | TETTI051-24 | *Synephippius obvius* |  | 42.371 | 0.853 | Spain | Lleida, Perves |
| T091634 | Bradyporinae | PQ568740 | TETTI279-24 | *Uromenus agarenus* |  | 36.127 | -5.706 | Spain | Cádiz, Tarifa, Puerto de Facinas |
| T092290 | Bradyporinae | PQ568802 | TETTI342-24 | *Uromenus brevicollis ibericus* |  | 37.334 | -6.79 | Spain | Huelva, Trigueros |
| T091633 | Bradyporinae | PQ568739 | TETTI278-24 | *Uromenus maroccanus* |  | 36.057 | -5.555 | Spain | Cádiz, Tarifa |
| T092045 | Bradyporinae | PQ568772 | TETTI311-24 | *Uromenus rugosicollis* |  | 41.653 | 2.687 | Spain | Barcelona, Santa Susanna |
| T092046 | Bradyporinae | PQ568773 | TETTI312-24 | *Uromenus rugosicollis* |  | 41.653 | 2.687 | Spain | Barcelona, Santa Susanna |
| T090427 | Bradyporinae | PQ568565 | TETTI104-24 | *Pycnogaster algecirensis* |  | 36.825 | -3.965 | Spain | Malaga, Cómpeta |
| T090152 | Bradyporinae | PQ568521 | TETTI060-24 | *Pycnogaster inermis* |  | 37.089 | -2.995 | Spain | Almería, Bayárcal, 7.2 km por pista al E del Puerto de la Ragua |
| T090170 | Bradyporinae | PQ568529 | TETTI068-24 | *Pycnogaster inermis* |  | 37.096 | -3.385 | Spain | Granada, Güejar Sierra, Sierra Nevada, Peñones de San Francisco |
| T090525 | Bradyporinae | PQ568580 | TETTI119-24 | *Pycnogaster jugicola* |  | 40.347 | -7.65 | Portugal | Guarda, Seia, Loriga, Serra da Estrela |
| T090324 | Bradyporinae | PQ568542 | TETTI081-24 | *Pycnogaster jugicola* |  | 40.98 | -3.799 | Spain | Madrid, Lozoya, Puerto de Navafría |
| T090323 | Bradyporinae | PQ568541 | TETTI080-24 | *Pycnogaster jugicola* |  | 40.842 | -3.835 | Spain | Madrid, Rascafría, Puerto de la Morcuera |
| INV06944 | Conocephalinae | OR974561 | IBIOR449-22 | *Conocephalus conocephalus* |  | 37.653 | -7.644 | Portugal | Beja, Mertola, Bombeira do Guadiana |
| INV06948 | Conocephalinae | OR974493 | IBIOR453-22 | *Conocephalus conocephalus* |  | 37.653 | -7.644 | Portugal | Beja, Mertola, Bombeira do Guadiana |
| INV06923 | Conocephalinae | OR974757 | IBIOR445-22 | *Conocephalus conocephalus* |  | 39.822 | -6.973 | Portugal | Castelo Branco, Idanha-a-Nova, Segura |
| INV06925 | Conocephalinae | OR974573 | IBIOR446-22 | *Conocephalus conocephalus* |  | 39.822 | -6.973 | Portugal | Castelo Branco, Idanha-a-Nova, Segura |
| T091674 | Conocephalinae | PQ568750 | TETTI289-24 | *Conocephalus conocephalus* |  | 39.302 | -8.801 | Portugal | Santarem, Abitureiras |
| T091675 | Conocephalinae | PQ568751 | TETTI290-24 | *Conocephalus conocephalus* |  | 39.302 | -8.801 | Portugal | Santarem, Abitureiras |
| **ID** | **Subfamily** | **Genbank** | **BOLD code** | **Species** | **Introgression Event** | **Latitude** | **Longitude** | **Country** | **Locality** |
| INV05569 | Conocephalinae | OR974496 | IBIOR220-22 | *Conocephalus conocephalus* |  | 38.566 | -9.034 | Portugal | Setubal, Sesimbra, Parque Ecologico da Varzea da Quinta do Conde |
| INV05570 | Conocephalinae | OR974443 | IBIOR221-22 | *Conocephalus conocephalus* |  | 38.566 | -9.034 | Portugal | Setubal, Sesimbra, Parque Ecologico da Varzea da Quinta do Conde |
| INV09078 | Conocephalinae | OR974579 | IBIOR513-22 | *Conocephalus fuscus* |  | 37.677 | -7.849 | Portugal | Beja, Mertola, Herdade de Alagaes |
| INV05583 | Conocephalinae | OR974609 | IBIOR234-22 | *Conocephalus fuscus* |  | 38.209 | -7.306 | Portugal | Beja, Moura, Povoa de Sao Miguel |
| INV06634 | Conocephalinae | OR974829 | IBIOR435-22 | *Conocephalus fuscus* |  | 41.349 | -7.290 | Portugal | Bragança, Mirandela, Abreiro |
| T091676 | Conocephalinae | PQ568752 | TETTI291-24 | *Conocephalus fuscus* |  | 39.302 | -8.801 | Portugal | Santarem, Abitureiras |
| TTT157 | Conocephalinae | PQ568824 | TETTI364-24 | *Conocephalus fuscus* |  | 40.694 | -4.372 | Spain | Segovia, El Espinar, río Voltoya |
| TTT282 | Conocephalinae | PQ568846 | TETTI386-24 | *Conocephalus fuscus* |  | 41.971 | -6.385 | Spain | Zamora, Codesal |
| TTT283 | Conocephalinae | PQ568847 | TETTI387-24 | *Conocephalus fuscus* |  | 41.971 | -6.385 | Spain | Zamora, Codesal |
| TTT276 | Conocephalinae | PQ568844 | TETTI384-24 | *Conocephalus fuscus* |  | 42.106 | -6.406 | Spain | Zamora, Utrera de la Encomienda |
| INV04089 | Conocephalinae | OR974529 | IBIOR164-19 | *Ruspolia nitidula* |  | 40.385 | -7.705 | Portugal | Guarda, Seia, Central Hidroelectrica da Ponte de Jugais |
| INV04086 | Conocephalinae | OR974782 | IBIOR163-19 | *Ruspolia nitidula* |  | 41.303 | -8.651 | Portugal | Porto, Vila do Conde, Canidelo |
| T091680 | Conocephalinae | PQ568753 | TETTI292-24 | *Ruspolia nitidula* |  | 39.302 | -8.801 | Portugal | Santarem, Abitureiras |
| INV02561 | Conocephalinae | OR974654 | IBIOR119-17 | *Ruspolia nitidula* |  | 38.566 | -9.034 | Portugal | Setubal, Sesimbra, Quinta do Conde |
| T090181 | Conocephalinae | PQ568530 | TETTI069-24 | *Ruspolia nitidula* |  | 42.519 | -9.025 | Spain | A Coruña, Aguiño, Santa Uxía de Ribeira |
| JGR194 | Conocephalinae | PQ568482 | TETTI021-24 | *Ruspolia nitidula* |  | 40.063 | -3.531 | Spain | Madrid, Aranjuez |
| INV08776 | Meconematidae | OR974639 | IBIOR510-22 | *Cyrtaspis scutata* |  | 38.773 | -9.465 | Portugal | Lisboa, Sintra, Near Estudante da Peninha |
| INV07949 | Meconematidae | OR974541 | IBIOR493-22 | *Cyrtaspis scutata* |  | 41.328 | -8.673 | Portugal | Porto, Vila do Conde, Campus Agrario de Vairao |
| INV09030 | Meconematidae | OR974656 | IBIOR512-22 | *Cyrtaspis scutata* |  | 38.475 | -8.994 | Portugal | Setubal, Setubal, Convento da Arrabida |
| INV04094 | Meconematidae | OR974507 | IBIOR165-19 | *Meconema thalassinum* |  | 41.844 | -6.862 | Portugal | Bragança, Bragança, Gondesende, Cepo Verde |
| INV03944 | Meconematidae | OR974750 | IBIOR160-19 | *Meconema thalassinum* |  | 40.419 | -7.522 | Portugal | Guarda, Manteigas, Carvalheira |
| INV03732 | Meconematidae | OR974440 | IBIOR159-19 | *Meconema thalassinum* |  | 41.328 | -8.673 | Portugal | Porto, Vila do Conde, Campus Agrario de Vairao |
| T090317 | Meconematinae | PQ568539 | TETTI078-24 | *Canariola* cf. *emarginata* |  | 37.683 | -2.189 | Spain | Almería, Maria, Sierra de Maria, 1700m |
| T090411 | Meconematinae | PQ568561 | TETTI100-24 | *Cyrtaspis scutata* |  | 43.554 | -6.093 | Spain | Asturias, Reborio, Muros de Nalón |
| T092064 | Meconematinae | PQ568778 | TETTI317-24 | *Cyrtaspis tuberculata* |  | 37.08 | -6.686 | Spain | Huelva, Almonte, Cuesta Maneli |
| T090379 | Meconematinae | PQ568554 | TETTI093-24 | *Meconema thalassinum* |  | 43.554 | -6.093 | Spain | Asturias, Reborio, Muros de Nalón |
| T091039 | Phaneropterinae | PQ568659 | TETTI198-24 | *Barbitistes fischeri* |  | 42.22 | 2.174 | Spain | Girona, Campdevanol |
| T091215 | Phaneropterinae | PQ568679 | TETTI218-24 | *Barbitistes fischeri* |  | 40.671 | -2.135 | Spain | Guadalajara, Peñalén |
| T092055 | Phaneropterinae | PQ568776 | TETTI315-24 | *Barbitistes fischeri* |  | 42.091 | 1.526 | Spain | Lleida, Lladurs |
| T092173 | Phaneropterinae | PQ568792 | TETTI331-24 | *Isophya pyrenaea* |  | 42.073 | 2.408 | Spain | Barcelona, Santa Maria de Corcó, Serra de Cabrera |
| T092173 | Phaneropterinae | PQ568792 | TETTI332-24 | *Isophya pyrenaea* |  | 42.073 | 2.408 | Spain | Barcelona, Santa Maria de Corcó, Serra de Cabrera |
| INV05562 | Phaneropterinae | OR974467 | IBIOR213-22 | *Leptophyes punctatissima* |  | 41.831 | -7.943 | Portugal | Vila Real, Montalegre, Pitoes das Junias, near Mosteiro |
| T091501 | Phaneropterinae | PQ568724 | TETTI263-24 | *Leptophyes punctatissima* |  | 42.701 | -3.276 | Spain | Burgos, Oña, La Aldea |
| T091927 | Phaneropterinae | PQ568759 | TETTI298-24 | *Leptophyes punctatissima* |  | 42.101 | 1.645 | Spain | Lleida, Navès |
| **ID** | **Subfamily** | **Genbank** | **BOLD code** | **Species** | **Introgression Event** | **Latitude** | **Longitude** | **Country** | **Locality** |
| T092042 | Phaneropterinae | PQ568771 | TETTI310-24 | *Metaplastes pulchripennis* |  | 41.653 | 2.687 | Spain | Barcelona, Santa Susanna |
| T092174 | Phaneropterinae | PQ568793 | TETTI333-24 | *Metaplastes pulchripennis* |  | 41.638 | 2.487 | Spain | Barcelona, Vilalba Sasserra, el Corredor, Serra del Montnegre |
| T091055 | Phaneropterinae | PQ568668 | TETTI207-24 | *Odontura aspericauda* |  | 36.852 | -2.161 | Spain | Almería, Níjar, Serrata de Nijar |
| T092073 | Phaneropterinae | PQ568782 | TETTI321-24 | *Odontura aspericauda* |  | 36.722 | -4.965 | Spain | Málaga, Yunquera, Sierra de las Nieves, Puerto del Saucillo |
| T092285 | Phaneropterinae | PQ568799 | TETTI339-24 | *Odontura aspericauda* |  | 37.904 | -5.809 | Spain | Sevilla, Cazalla de la Sierra |
| INV00117 | Phaneropterinae | OR974651 | IBIOR050-17 | *Odontura glabricauda* |  | 37.746 | -8.199 | Portugal | Beja, Castro Verde, Casevel |
| INV02515 | Phaneropterinae | OR974587 | IBIOR080-17 | *Odontura glabricauda* |  | 37.719 | -7.812 | Portugal | Beja, Mertola, Alcaria Ruiva |
| T090429 | Phaneropterinae | PQ568566 | TETTI105-24 | *Odontura glabricauda* |  | 37.537 | -7.599 | Portugal | Beja, Sedas |
| INV06518 | Phaneropterinae | OR974459 | IBIOR419-22 | *Odontura glabricauda* |  | 39.680 | -7.098 | Portugal | Castelo Branco, Idanha-a-Nova, Herdade da Poupa |
| INV06520 | Phaneropterinae | OR974804 | IBIOR420-22 | *Odontura glabricauda* |  | 39.680 | -7.098 | Portugal | Castelo Branco, Idanha-a-Nova, Herdade da Poupa |
| INV08771 | Phaneropterinae | OR974664 | IBIOR509-22 | *Odontura glabricauda* |  | 39.469 | -8.851 | Portugal | Santarem, Santarem, Alcanede |
| T090861 | Phaneropterinae | PQ568648 | TETTI187-24 | *Odontura glabricauda* |  | 38.062 | -8.434 | Portugal | Setúbal, Azinheira dos Barros |
| T091428 | Phaneropterinae | PQ568712 | TETTI251-24 | *Odontura glabricauda* |  | 39.214 | -6.127 | Spain | Cáceres, Montánchez |
| INV05587 | Phaneropterinae | OR974608 | IBIOR238-22 | *Odontura macphersoni* |  | 40.350 | -7.549 | Portugal | Guarda, Manteigas, Parque Natural da Serra da Estrela |
| INV05588 | Phaneropterinae | OR974456 | IBIOR239-22 | *Odontura macphersoni* |  | 40.350 | -7.549 | Portugal | Guarda, Manteigas, Parque Natural da Serra da Estrela |
| T090929 | Phaneropterinae | PQ568654 | TETTI193-24 | *Odontura macphersoni* |  | 40.271 | -5.251 | Spain | Ávila, Navalperal de Tormes, Sierra de Gredos |
| T091935 | Phaneropterinae | PQ568761 | TETTI300-24 | *Odontura macphersoni* |  | 42.206 | -6.546 | Spain | León, Truchas, Falda O Pico Vizcodillo |
| T090377 | Phaneropterinae | PQ568553 | TETTI092-24 | *Phaneroptera falcata* |  | 43.336 | -4.338 | Spain | Cantabria, Valdáliga, Vallines |
| T090773 | Phaneropterinae | PQ568620 | TETTI159-24 | *Phaneroptera falcata* |  | 42.824 | 0.794 | Spain | Lleida, Canejan, San Juan de Toran |
| INV11453 | Phaneropterinae | OR974449 | IBIOR555-22 | *Phaneroptera nana* |  | 37.677 | -7.849 | Portugal | Beja, Mertola, Herdade de Alagaes |
| INV09088 | Phaneropterinae | OR974469 | IBIOR515-22 | *Phaneroptera nana* |  | 37.803 | -7.635 | Portugal | Beja, Mertola, Pulo do Lobo |
| INV10234 | Phaneropterinae | OR974481 | IBIOR520-22 | *Phaneroptera nana* |  | 41.766 | -6.902 | Portugal | Bragança, Bragança, Carrazedo |
| INV10271 | Phaneropterinae | OR974586 | IBIOR522-22 | *Phaneroptera nana* |  | 41.921 | -6.575 | Portugal | Bragança, Bragança, Guadramil |
| INV06063 | Phaneropterinae | OR974764 | IBIOR408-22 | *Phaneroptera nana* |  | 41.348 | -7.306 | Portugal | Bragança, Mirandela, Abreiro |
| INV06629 | Phaneropterinae | OR974655 | IBIOR433-22 | *Phaneroptera nana* |  | 41.349 | -7.290 | Portugal | Bragança, Mirandela, Abreiro |
| INV06558 | Phaneropterinae | OR974510 | IBIOR424-22 | *Phaneroptera nana* |  | 41.315 | -7.220 | Portugal | Bragança, Vila Flor, Freixiel |
| INV06560 | Phaneropterinae | OR974758 | IBIOR425-22 | *Phaneroptera nana* |  | 41.315 | -7.220 | Portugal | Bragança, Vila Flor, Freixiel |
| INV04008 | Phaneropterinae | OR974667 | IBIOR341-22 | *Phaneroptera nana* |  | 41.909 | -6.933 | Portugal | Bragança, Vinhais, Dine |
| INV04010 | Phaneropterinae | OR974798 | IBIOR344-22 | *Phaneroptera nana* |  | 41.909 | -6.933 | Portugal | Bragança, Vinhais, Dine |
| INV04021 | Phaneropterinae | OR974799 | IBIOR345-22 | *Phaneroptera nana* |  | 41.909 | -6.933 | Portugal | Bragança, Vinhais, Dine |
| INV04042 | Phaneropterinae | OR974444 | IBIOR349-22 | *Phaneroptera nana* |  | 41.867 | -6.983 | Portugal | Bragança, Vinhais, Paçó, Barragem de Prada |
| INV03968 | Phaneropterinae | OR974614 | IBIOR334-22 | *Phaneroptera nana* |  | 40.385 | -7.705 | Portugal | Guarda, Seia, Hydroelectric power plant of Ponte de Jugais |
| INV03978 | Phaneropterinae | OR974650 | IBIOR335-22 | *Phaneroptera nana* |  | 40.385 | -7.705 | Portugal | Guarda, Seia, Hydroelectric power plant of Ponte de Jugais |
| INV02559 | Phaneropterinae | OR974643 | IBIOR117-17 | *Phaneroptera nana* |  | 38.548 | -9.014 | Portugal | Setubal, Setubal, Brejos dos Clerigos |
| **ID** | **Subfamily** | **Genbank** | **BOLD code** | **Species** | **Introgression Event** | **Latitude** | **Longitude** | **Country** | **Locality** |
| INV10339 | Phaneropterinae | OR974813 | IBIOR528-22 | *Phaneroptera nana* |  | 41.838 | -8.732 | Portugal | Viana do Castelo, Caminha, Mosteiro de Sao Joao de Arga |
| INV06087 | Phaneropterinae | OR974416 | IBIOR412-22 | *Phaneroptera nana* |  | 41.284 | -7.396 | Portugal | Vila Real, Alijo, Amieiro |
| T091371 | Phaneropterinae | PQ568704 | TETTI243-24 | *Phaneroptera nana* |  | 42.717 | -3.343 | Spain | Burgos, Barcina de los Montes |
| T091858 | Phaneropterinae | PQ568757 | TETTI296-24 | *Phaneroptera nana* |  | 40.954 | -2.04 | Spain | Guadalajara, Aragoncillo |
| T091961 | Phaneropterinae | PQ568765 | TETTI304-24 | *Phaneroptera nana* |  | 37.051 | -6.625 | Spain | Huelva, Almonte, Doñana |
| T092293 | Phaneropterinae | PQ568805 | TETTI345-24 | *Phaneroptera nana* |  | 37.92 | -5.588 | Spain | Sevilla, Constantina, Cerro del Negrillo |
| INV06918 | Phaneropterinae | OR974512 | IBIOR444-22 | *Phaneroptera sparsa* |  | 39.822 | -6.973 | Portugal | Castelo Branco, Idanha-a-Nova, Segura |
| INV06926 | Phaneropterinae | OR974518 | IBIOR447-22 | *Phaneroptera sparsa* |  | 39.822 | -6.973 | Portugal | Castelo Branco, Idanha-a-Nova, Segura |
| INV07540 | Phaneropterinae | OR974619 | IBIOR472-22 | *Phaneroptera sparsa* |  | 37.103 | -7.963 | Portugal | Faro, Faro, Santa Barbara de Nexe |
| INV07543 | Phaneropterinae | OR974771 | IBIOR474-22 | *Phaneroptera sparsa* |  | 37.197 | -7.922 | Portugal | Faro, Sao Bras de Alportel, Sao Bras de Alportel |
| INV02520 | Phaneropterinae | OR974792 | IBIOR085-17 | *Tylopsis lilifolia* |  | 37.869 | -8.118 | Portugal | Beja, Aljustrel, Aljustrel |
| INV02521 | Phaneropterinae | OR974422 | IBIOR086-17 | *Tylopsis lilifolia* |  | 37.880 | -8.141 | Portugal | Beja, Aljustrel, Aljustrel |
| INV00140 | Phaneropterinae | OR974552 | IBIOR064-17 | *Tylopsis lilifolia* |  | 37.748 | -7.858 | Portugal | Beja, Mertola, Alcaria Ruiva |
| INV00141 | Phaneropterinae | OR974797 | IBIOR065-17 | *Tylopsis lilifolia* |  | 37.731 | -7.858 | Portugal | Beja, Mertola, Alcaria Ruiva |
| INV10591 | Phaneropterinae | OR974502 | IBIOR533-22 | *Tylopsis lilifolia* |  | 37.653 | -7.644 | Portugal | Beja, Mertola, Bombeira do Guadiana |
| T091687 | Phaneropterinae | PQ568755 | TETTI294-24 | *Tylopsis lilifolia* |  | 38.12 | -8.787 | Portugal | Setúbal, Santiago do Cacém |
| T092013 | Phaneropterinae | PQ568770 | TETTI309-24 | *Tylopsis lilifolia* |  | 41.512 | 2.088 | Spain | Barcelona, Sant Quirze del Vallès |
| T091977 | Phaneropterinae | PQ568767 | TETTI306-24 | *Tylopsis lilifolia* |  | 41.873 | 2.501 | Spain | Girona, Sant Hilari Sacalm |
| T090415 | Saginae | PQ568562 | TETTI101-24 | *Saga pedo* |  | 40.171 | -2.231 | Spain | Cuenca, 2 Km al NO de Tondos |
| T090901 | Saginae | PQ568649 | TETTI188-24 | *Saga pedo* |  | - | - | Spain | Madrid, Valdemanco |
| T091615 | Tettigoniinae | PQ568738 | TETTI277-24 | *Amphiestris baetica* |  | 36.891 | -5.9 | Spain | Sevilla, Lebrija |
| T091645 | Tettigoniinae | PQ568743 | TETTI282-24 | *Amphiestris baetica* |  | 37.475 | -6.088 | Spain | Sevilla, Salteras |
| T091048 | Tettigoniinae | PQ568666 | TETTI205-24 | *Antaxius chopardi* |  | 42.281 | 2.201 | Spain | Girona, Ribes de Freser, pla de la Jaça, el Taga |
| T091928 | Tettigoniinae | PQ568760 | TETTI299-24 | *Antaxius chopardi* |  | 42.101 | 1.645 | Spain | Lleida, Navès |
| INV05548 | Tettigoniinae | OR974719 | IBIOR199-22 | *Antaxius florezi* |  | 42.001 | -8.289 | Portugal | Viana do Castelo, Melgaco, Gave, near road EM503 |
| INV05549 | Tettigoniinae | OR974562 | IBIOR200-22 | *Antaxius florezi* |  | 42.001 | -8.289 | Portugal | Viana do Castelo, Melgaco, Gave, near road EM503 |
| T090343 | Tettigoniinae | PQ568544 | TETTI083-24 | *Antaxius florezi* |  | 43.405 | -4.99 | Spain | Asturias, Llanes, Alto del Torno |
| T090370 | Tettigoniinae | PQ568549 | TETTI088-24 | *Antaxius florezi* |  | 43.05 | -6.11 | Spain | Asturias, Somiedo, Lagos de Salienza |
| T090371 | Tettigoniinae | PQ568550 | TETTI089-24 | *Antaxius florezi* |  | 43.05 | -6.11 | Spain | Asturias, Somiedo, Lagos de Salienza |
| T090372 | Tettigoniinae | PQ568551 | TETTI090-24 | *Antaxius florezi* |  | 43.05 | -6.11 | Spain | Asturias, Somiedo, Lagos de Salienza |
| T090373 | Tettigoniinae | OR187097 | - | *Antaxius florezi* |  | 43.050 | -6.110 | Spain | Asturias, Somiedo, Lagos de Salienza |
| T090514 | Tettigoniinae | PQ568575 | TETTI114-24 | *Antaxius florezi* |  | 42.992 | -6.411 | Spain | León, Villablino, Leitariegos |
| T090515 | Tettigoniinae | PQ568576 | TETTI115-24 | *Antaxius florezi* |  | 42.992 | -6.411 | Spain | León, Villablino, Leitariegos |
| T090516 | Tettigoniinae | PQ568577 | TETTI116-24 | *Antaxius florezi* |  | 42.992 | -6.411 | Spain | León, Villablino, Leitariegos |
| **ID** | **Subfamily** | **Genbank** | **BOLD code** | **Species** | **Introgression Event** | **Latitude** | **Longitude** | **Country** | **Locality** |
| T090517 | Tettigoniinae | PQ568578 | TETTI117-24 | *Antaxius florezi* |  | 42.992 | -6.411 | Spain | León, Villablino, Leitariegos |
| T090096 | Tettigoniinae | PQ568514 | TETTI053-24 | *Antaxius florezi* |  | 42.522 | -8.132 | Spain | Ourense, O Irixo |
| TTT383 | Tettigoniinae | PQ568860 | TETTI400-24 | *Antaxius hispanicus* |  | 42.582 | 0.546 | Spain | Huesca, Benasque, Cerler |
| TTT384 | Tettigoniinae | PQ568861 | TETTI401-24 | *Antaxius hispanicus* |  | 42.582 | 0.546 | Spain | Huesca, Benasque, Cerler |
| TTT385 | Tettigoniinae | PQ568862 | TETTI402-24 | *Antaxius hispanicus* |  | 42.582 | 0.546 | Spain | Huesca, Benasque, Cerler |
| T090312 | Tettigoniinae | PQ568534 | TETTI073-24 | *Antaxius hispanicus* |  | 42.487 | 0.875 | Spain | Lleida, La Vall de Boí, Estacion de esqui Boí Taüll |
| T090148 | Tettigoniinae | PQ568517 | TETTI056-24 | *Antaxius kraussii* |  | 37.085 | -2.924 | Spain | Almería, Láujar de Andarax |
| T090149 | Tettigoniinae | PQ568518 | TETTI057-24 | *Antaxius kraussii* |  | 37.085 | -2.924 | Spain | Almería, Láujar de Andarax |
| T090150 | Tettigoniinae | PQ568519 | TETTI058-24 | *Antaxius kraussii* |  | 37.085 | -2.924 | Spain | Almería, Láujar de Andarax |
| T090151 | Tettigoniinae | PQ568520 | TETTI059-24 | *Antaxius kraussii* |  | 37.085 | -2.924 | Spain | Almería, Láujar de Andarax |
| T090316 | Tettigoniinae | PQ568538 | TETTI077-24 | *Antaxius kraussii* |  | 37.096 | -2.998 | Spain | Granada, Dólar, Sierra Nevada, El Chullo, 2700m |
| T091280 | Tettigoniinae | OR187080 | - | *Antaxius oretanus* |  | 39.472 | -5.395 | Spain | Cáceres, Navezuelas, Pico Villuercas |
| T091281 | Tettigoniinae | OR187081 | - | *Antaxius oretanus* |  | 39.472 | -5.395 | Spain | Cáceres, Navezuelas, Pico Villuercas |
| T091276 | Tettigoniinae | OR187078 | - | *Antaxius oretanus* |  | 39.574 | -5.348 | Spain | Cáceres, Villar del Pedroso, Pico Carbonero |
| T091277 | Tettigoniinae | OR187079 | - | *Antaxius oretanus* |  | 39.574 | -5.348 | Spain | Cáceres, Villar del Pedroso, Pico Carbonero |
| T090620 | Tettigoniinae | OR187056 | - | *Antaxius oretanus* |  | 39.524 | -4.358 | Spain | Toledo, San Pablo de los Montes, Puerto del Robledillo |
| T090621 | Tettigoniinae | OR187057 | - | *Antaxius oretanus* |  | 39.524 | -4.358 | Spain | Toledo, San Pablo de los Montes, Puerto del Robledillo |
| T090622 | Tettigoniinae | OR187058 | - | *Antaxius oretanus* |  | 39.524 | -4.358 | Spain | Toledo, San Pablo de los Montes, Puerto del Robledillo |
| T090623 | Tettigoniinae | OR187059 | - | *Antaxius oretanus* |  | 39.524 | -4.358 | Spain | Toledo, San Pablo de los Montes, Puerto del Robledillo |
| INV04034 | Tettigoniinae | OR974576 | IBIOR347-22 | *Antaxius spinibrachius* |  | 41.905 | -6.776 | Portugal | Bragança, Bragança, Franca |
| INV04036 | Tettigoniinae | OR974417 | IBIOR348-22 | *Antaxius spinibrachius* |  | 41.905 | -6.776 | Portugal | Bragança, Bragança, Franca |
| T090490 | Tettigoniinae | PQ568572 | TETTI111-24 | *Antaxius spinibrachius* |  | 41.436 | -6.999 | Portugal | Bragança, Macedo de Cavaleiros, Serra de Bornes |
| T090491 | Tettigoniinae | PQ568573 | TETTI112-24 | *Antaxius spinibrachius* |  | 41.436 | -6.999 | Portugal | Bragança, Macedo de Cavaleiros, Serra de Bornes |
| T090492 | Tettigoniinae | OR187051 | - | *Antaxius spinibrachius* |  | 41.436 | -6.999 | Portugal | Bragança, Macedo de Cavaleiros, Serra de Bornes |
| T090493 | Tettigoniinae | OR187052 | - | *Antaxius spinibrachius* |  | 41.436 | -6.999 | Portugal | Bragança, Macedo de Cavaleiros, Serra de Bornes |
| INV04088 | Tettigoniinae | OR974791 | IBIOR352-22 | *Antaxius spinibrachius* |  | 41.597 | -6.559 | Portugal | Bragança, Vimioso, Vimioso N6 |
| INV01297 | Tettigoniinae | OR974748 | IBIOR284-22 | *Antaxius spinibrachius* |  | 40.315 | -7.558 | Portugal | Castelo Branco, Covilha, Near Penhas da Saude |
| T090539 | Tettigoniinae | PQ568585 | TETTI124-24 | *Antaxius spinibrachius* |  | 40.315 | -7.577 | Portugal | Castelo Branco, Covilhã, Serra da Estrela |
| T090540 | Tettigoniinae | PQ568586 | TETTI125-24 | *Antaxius spinibrachius* |  | 40.315 | -7.577 | Portugal | Castelo Branco, Covilhã, Serra da Estrela |
| INV04000 | Tettigoniinae | OR974743 | IBIOR339-22 | *Antaxius spinibrachius* |  | 40.419 | -7.522 | Portugal | Guarda, Manteigas, Carvalheira |
| INV04001 | Tettigoniinae | OR974445 | IBIOR340-22 | *Antaxius spinibrachius* |  | 40.419 | -7.522 | Portugal | Guarda, Manteigas, Carvalheira |
| INV03948 | Tettigoniinae | OR974810 | IBIOR332-22 | *Antaxius spinibrachius* |  | 40.385 | -7.705 | Portugal | Guarda, Seia, Hydroelectric power plant of Ponte de Jugais |
| INV03993 | Tettigoniinae | OR974712 | IBIOR336-22 | *Antaxius spinibrachius* |  | 40.393 | -7.601 | Portugal | Guarda, Seia, Vale do Rossim |
| INV03994 | Tettigoniinae | OR974693 | IBIOR337-22 | *Antaxius spinibrachius* |  | 40.393 | -7.601 | Portugal | Guarda, Seia, Vale do Rossim |
| **ID** | **Subfamily** | **Genbank** | **BOLD code** | **Species** | **Introgression Event** | **Latitude** | **Longitude** | **Country** | **Locality** |
| INV07171 | Tettigoniinae | OR974688 | IBIOR567-22 | *Antaxius spinibrachius* |  | 41.280 | -7.400 | Portugal | Vila Real, Alijo, Amieiro |
| INV07172 | Tettigoniinae | OR974426 | IBIOR568-22 | *Antaxius spinibrachius* |  | 41.280 | -7.400 | Portugal | Vila Real, Alijo, Amieiro |
| INV07174 | Tettigoniinae | OR974824 | IBIOR569-22 | *Antaxius spinibrachius* |  | 41.280 | -7.400 | Portugal | Vila Real, Alijo, Amieiro |
| T091166 | Tettigoniinae | OR187071 | - | *Antaxius spinibrachius* |  | 41.561 | -7.517 | Portugal | Vila Real, Chaves, Serra da Padrela |
| T091167 | Tettigoniinae | PQ568676 | TETTI215-24 | *Antaxius spinibrachius* |  | 41.561 | -7.517 | Portugal | Vila Real, Chaves, Serra da Padrela |
| INV05550 | Tettigoniinae | OR974641 | IBIOR201-22 | *Antaxius spinibrachius* |  | 41.831 | -7.943 | Portugal | Vila Real, Montalegre, Pitoes das Junias, near Mosteiro |
| INV05551 | Tettigoniinae | OR974472 | IBIOR202-22 | *Antaxius spinibrachius* |  | 41.831 | -7.943 | Portugal | Vila Real, Montalegre, Pitoes das Junias, near Mosteiro |
| T090390 | Tettigoniinae | OR187044 | - | *Antaxius spinibrachius* |  | 40.336 | -1.761 | Spain | Cuenca, Tragacete |
| T090391 | Tettigoniinae | PQ568558 | TETTI097-24 | *Antaxius spinibrachius* |  | 40.336 | -1.761 | Spain | Cuenca, Tragacete |
| T091372 | Tettigoniinae | OR187088 | - | *Antaxius spinibrachius* |  | 42.891 | -4.268 | Spain | Palencia, Vallejo de Orbó |
| T091373 | Tettigoniinae | OR187089 | - | *Antaxius spinibrachius* |  | 42.891 | -4.268 | Spain | Palencia, Vallejo de Orbó |
| T091374 | Tettigoniinae | OR187090 | - | *Antaxius spinibrachius* |  | 42.891 | -4.268 | Spain | Palencia, Vallejo de Orbó |
| TTT377 | Tettigoniinae | PQ568856 | TETTI396-24 | *Bicolorana bicolor* |  | 42.823 | 0.798 | Spain | Lleida, Canejan, Sant Joan de Toran |
| TTT378 | Tettigoniinae | PQ568857 | TETTI397-24 | *Bicolorana bicolor* |  | 42.823 | 0.798 | Spain | Lleida, Canejan, Sant Joan de Toran |
| T090667 | Tettigoniinae | PQ568597 | TETTI136-24 | *Ctenodecticus granatensis* |  | 37.606 | -4.035 | Spain | Jaen, Alcaudete, Sierra Ahillo |
| T090668 | Tettigoniinae | PQ568598 | TETTI137-24 | *Ctenodecticus granatensis* |  | 37.606 | -4.035 | Spain | Jaen, Alcaudete, Sierra Ahillo |
| T090669 | Tettigoniinae | PQ568599 | TETTI138-24 | *Ctenodecticus granatensis* |  | 37.606 | -4.035 | Spain | Jaen, Alcaudete, Sierra Ahillo |
| T090670 | Tettigoniinae | PQ568600 | TETTI139-24 | *Ctenodecticus granatensis* |  | 37.606 | -4.035 | Spain | Jaen, Alcaudete, Sierra Ahillo |
| T090526 | Tettigoniinae | PQ568581 | TETTI120-24 | *Ctenodecticus lusitanicus* |  | 40.347 | -7.65 | Portugal | Guarda, Loriga, Serra da Estrela |
| T090528 | Tettigoniinae | PQ568582 | TETTI121-24 | *Ctenodecticus lusitanicus* |  | 40.347 | -7.65 | Portugal | Guarda, Loriga, Serra da Estrela |
| T090529 | Tettigoniinae | PQ568583 | TETTI122-24 | *Ctenodecticus lusitanicus* |  | 40.347 | -7.65 | Portugal | Guarda, Loriga, Serra da Estrela |
| T090676 | Tettigoniinae | PQ568601 | TETTI140-24 | *Ctenodecticus major* |  | 36.946 | -3.406 | Spain | Granada, Soportújar, Sierra Nevada, Hoya del Nevazo |
| T090677 | Tettigoniinae | PQ568602 | TETTI141-24 | *Ctenodecticus major* |  | 36.946 | -3.406 | Spain | Granada, Soportújar, Sierra Nevada, Hoya del Nevazo |
| T090910 | Tettigoniinae | PQ568651 | TETTI190-24 | *Ctenodecticus masferreri* |  | 41.802 | 2.348 | Spain | Barcelona, El Brull, Serra del Montseny, Coll Formic |
| T090911 | Tettigoniinae | PQ568652 | TETTI191-24 | *Ctenodecticus masferreri* |  | 41.802 | 2.348 | Spain | Barcelona, El Brull, Serra del Montseny, Coll Formic |
| T090331 | Tettigoniinae | PQ568543 | TETTI082-24 | *Ctenodecticus pupulus* |  | 40.853 | -3.654 | Spain | Madrid, Valdemanco, Ctra Valdemanco-Cabanillas |
| T090374 | Tettigoniinae | PQ568552 | TETTI091-24 | *Ctenodecticus pupulus* |  | 40.853 | -3.654 | Spain | Madrid, Valdemanco, Ctra Valdemanco-Cabanillas |
| T091916 | Tettigoniinae | PQ568758 | TETTI297-24 | *Ctenodecticus pupulus* |  | 40.4 | -6.397 | Spain | Salamanca, Agallas, Puerto de Esperabán |
| T090658 | Tettigoniinae | PQ568593 | TETTI132-24 | *Ctenodecticus ramburi* |  | 38.051 | -2.555 | Spain | Granada, Huescar, Pinar de la Vidriera |
| T090659 | Tettigoniinae | PQ568594 | TETTI133-24 | *Ctenodecticus ramburi* |  | 38.051 | -2.555 | Spain | Granada, Huescar, Pinar de la Vidriera |
| T090660 | Tettigoniinae | PQ568595 | TETTI134-24 | *Ctenodecticus ramburi* |  | 38.051 | -2.555 | Spain | Granada, Huescar, Pinar de la Vidriera |
| T090661 | Tettigoniinae | PQ568596 | TETTI135-24 | *Ctenodecticus ramburi* |  | 38.051 | -2.555 | Spain | Granada, Huescar, Pinar de la Vidriera |
| T090314 | Tettigoniinae | PQ568536 | TETTI075-24 | *Ctenodecticus thymi* |  | 42.042 | 0.759 | Spain | Lleida, Sant Esteve de la Sarga, Sierra del Montsec d'Ares |
| T090315 | Tettigoniinae | PQ568537 | TETTI076-24 | *Ctenodecticus thymi* |  | 42.042 | 0.759 | Spain | Lleida, Sant Esteve de la Sarga, Sierra del Montsec d'Ares |
| **ID** | **Subfamily** | **Genbank** | **BOLD code** | **Species** | **Introgression Event** | **Latitude** | **Longitude** | **Country** | **Locality** |
| INV08116 | Tettigoniinae | OR974550 | IBIOR271-22 | *Decticus albifrons* |  | 37.700 | -8.100 | Portugal | Beja, Castro Verde, Castro Verde |
| INV00115 | Tettigoniinae | OR974549 | IBIOR049-17 | *Decticus albifrons* |  | 37.641 | -7.880 | Portugal | Beja, Castro Verde, Sao Marcos da Ataboeira |
| INV02508 | Tettigoniinae | OR974446 | IBIOR073-17 | *Decticus albifrons* |  | 37.669 | -7.806 | Portugal | Beja, Mertola, Sao Joao dos Caldeireiros |
| JGR072 | Tettigoniinae | PQ568469 | TETTI008-24 | *Decticus albifrons* |  | 40.395 | -4.002 | Spain | Madrid, Brunete |
| JGR205 | Tettigoniinae | PQ568486 | TETTI025-24 | *Decticus albifrons* |  | 40.346 | -4.108 | Spain | Madrid, Villanueva de Perales |
| T090422 | Tettigoniinae | PQ568563 | TETTI102-24 | *Decticus verrucivorus* |  | 42.26 | -2.974 | Spain | La Rioja, Ezcaray, Sierra de la Demanda |
| T090523 | Tettigoniinae | PQ568579 | TETTI118-24 | *Decticus verrucivorus* |  | 42.296 | -7.323 | Spain | Ourense, A Pobra de Trives, Cabeza de Manzaneda |
| INV02577 | Tettigoniinae | OR974700 | IBIOR133-17 | *Incertana decorata* |  | 39.180 | -9.046 | Portugal | Lisboa, Cadaval, Quartel |
| INV02578 | Tettigoniinae | OR974759 | IBIOR134-17 | *Incertana decorata* |  | 39.180 | -9.046 | Portugal | Lisboa, Cadaval, Quartel |
| T090948 | Tettigoniinae | PQ568656 | TETTI195-24 | *Incertana decorata* |  | 39.398 | -8.939 | Portugal | Santarém, Rio Maior, Serra do Aires |
| T090949 | Tettigoniinae | PQ568657 | TETTI196-24 | *Incertana decorata* |  | 39.398 | -8.939 | Portugal | Santarém, Rio Maior, Serra do Aires |
| T091041 | Tettigoniinae | PQ568661 | TETTI200-24 | *Incertana decorata* |  | 36.133 | -5.486 | Spain | Cádiz, Algeciras, via verde Algeciras-Cadiz |
| T091042 | Tettigoniinae | PQ568662 | TETTI201-24 | *Incertana decorata* |  | 36.133 | -5.486 | Spain | Cádiz, Algeciras, via verde Algeciras-Cadiz |
| INV02600 | Tettigoniinae | OR974461 | IBIOR190-22 | *Metrioptera ambigua* |  | 42.036 | -8.136 | Portugal | Viana do Castelo, Melgaco, Castro Laboreiro |
| INV05542 | Tettigoniinae | OR974767 | IBIOR193-22 | *Metrioptera ambigua* |  | 42.036 | -8.136 | Portugal | Viana do Castelo, Melgaco, Castro Laboreiro |
| T090423 | Tettigoniinae | PQ568564 | TETTI103-24 | *Metrioptera ambigua* |  | 42.609 | -7.187 | Spain | Lugo, Folgoso do Courel, Serra do Courel |
| TTT274 | Tettigoniinae | PQ568843 | TETTI383-24 | *Metrioptera ambigua* |  | 42.163 | -6.906 | Spain | Zamora, Porto de Sanabria |
| TTT379 | Tettigoniinae | PQ568858 | TETTI398-24 | *Metrioptera buyssoni* |  | 42.913 | 0.764 | France | Haute-Garonne, Boutx, Le Mourtis |
| TTT380 | Tettigoniinae | PQ568859 | TETTI399-24 | *Metrioptera buyssoni* |  | 42.913 | 0.764 | France | Haute-Garonne, Boutx, Le Mourtis |
| T091414 | Tettigoniinae | PQ568709 | TETTI248-24 | *Metrioptera buyssoni* |  | 42.819 | 0.811 | Spain | Lleida, Canejan, Sant Joan de Toran, Arriu Toran |
| T091500 | Tettigoniinae | PQ568723 | TETTI262-24 | *Metrioptera maritima* |  | 43.169 | -3.634 | Spain | Burgos, Espinosa de los Monteros |
| T091413 | Tettigoniinae | PQ568708 | TETTI247-24 | *Metrioptera maritima* |  | 43.405 | -3.602 | Spain | Cantabria, Hazas de Cesto |
| T091085 | Tettigoniinae | PQ568670 | TETTI209-24 | *Metrioptera maritima* |  | 43.338 | -4.133 | Spain | Cantabria, Reocín, Quijas |
| T092728 | Tettigoniinae | PQ568806 | TETTI346-24 | *Metrioptera saussuriana* |  | 42.546 | 1.708 | Andorra | Encamp |
| T090225 | Tettigoniinae | PQ568531 | TETTI070-24 | *Metrioptera saussuriana* |  | 43.605 | 2.974 | France | Hérault, Rosis, Mont de Caroux |
| T090228 | Tettigoniinae | PQ568532 | TETTI071-24 | *Metrioptera saussuriana* |  | 43.605 | 2.974 | France | Hérault, Rosis, Mont de Caroux |
| T090229 | Tettigoniinae | PQ568533 | TETTI072-24 | *Metrioptera saussuriana* |  | 43.605 | 2.974 | France | Hérault, Rosis, Mont de Caroux |
| T091415 | Tettigoniinae | PQ568710 | TETTI249-24 | *Metrioptera saussuriana* | # *M. buyssoni* | 42.76 | 1.058 | Spain | Lleida, Alt Àneu, Bonabé, prats de Clavera |
| T091602 | Tettigoniinae | PQ568734 | TETTI273-24 | *Montana carpetana* |  | 38.77 | -5.515 | Spain | Badajoz, Castuera |
| T091046 | Tettigoniinae | PQ568664 | TETTI203-24 | *Montana carpetana* |  | 41.112 | -6.239 | Spain | Salamanca, Puertas, El Gróo |
| T091047 | Tettigoniinae | PQ568665 | TETTI204-24 | *Montana carpetana* |  | 41.112 | -6.239 | Spain | Salamanca, Puertas, El Gróo |
| TTT307 | Tettigoniinae | PQ568848 | TETTI388-24 | *Pholidoptera griseoaptera* |  | 42.969 | 0.725 | France | Haute-Garonne, Arguenos |
| T090908 | Tettigoniinae | PQ568650 | TETTI189-24 | *Pholidoptera griseoaptera* |  | 41.76 | 2.46 | Spain | Barcelona, Fogars de Montclús, Serra del Montseny |
| TTT369 | Tettigoniinae | PQ568853 | TETTI393-24 | *Pholidoptera griseoaptera* |  | 42.769 | 0.662 | Spain | Lleida, Bossòst, El Portillón |
| **ID** | **Subfamily** | **Genbank** | **BOLD code** | **Species** | **Introgression Event** | **Latitude** | **Longitude** | **Country** | **Locality** |
| INV00122 | Tettigoniinae | OR974752 | IBIOR053-17 | *Platycleis affinis* |  | 37.719 | -7.933 | Portugal | Beja, Castro Verde, Sao Marcos da Ataboeira |
| INV02516 | Tettigoniinae | OR974816 | IBIOR081-17 | *Platycleis affinis* |  | 37.785 | -8.277 | Portugal | Beja, Ourique, Conceicao |
| TTT032 | Tettigoniinae | PQ568810 | TETTI350-24 | *Platycleis affinis* |  | 40.926 | -3.435 | Spain | Guadalajara, 3 km NW Alpedrete de la Sierra |
| JGR070 | Tettigoniinae | PQ568467 | TETTI006-24 | *Platycleis affinis* |  | 40.395 | -4.002 | Spain | Madrid, Brunete |
| JGR295 | Tettigoniinae | PQ568498 | TETTI037-24 | *Platycleis affinis* |  | 40.693 | -4.049 | Spain | Madrid, Collado Mediano |
| JGR035 | Tettigoniinae | PQ568463 | TETTI002-24 | *Platycleis affinis* |  | 40.678 | -3.782 | Spain | Madrid, Colmenar Viejo |
| JGR176 | Tettigoniinae | PQ568478 | TETTI017-24 | *Platycleis affinis* |  | 40.551 | -3.549 | Spain | Madrid, Paracuellos de Jarama |
| JGR171 | Tettigoniinae | PQ568476 | TETTI015-24 | *Platycleis affinis* |  | 40.417 | -4.059 | Spain | Madrid, Quijorna |
| JGR267 | Tettigoniinae | PQ568497 | TETTI036-24 | *Platycleis affinis* |  | 40.595 | -4.243 | Spain | Madrid, Santa María de la Alameda |
| JGR170 | Tettigoniinae | PQ568475 | TETTI014-24 | *Platycleis affinis* |  | 40.44 | -4.018 | Spain | Madrid, Villanueva de la Cañada |
| TTT333 | Tettigoniinae | PQ568850 | TETTI390-24 | *Platycleis albopunctata* | # *Pl. intermedia* | 42.969 | 0.725 | Francia | Haute-Garonne, Arguenos |
| INV06511 | Tettigoniinae | OR974815 | IBIOR417-22 | *Platycleis albopunctata* |  | 41.315 | -7.220 | Portugal | Bragança, Vila Flor, Freixiel |
| INV07110 | Tettigoniinae | OR974626 | IBIOR458-22 | *Platycleis albopunctata* |  | 41.315 | -7.220 | Portugal | Bragança, Vila Flor, Freixiel |
| INV03998 | Tettigoniinae | OR974784 | IBIOR338-22 | *Platycleis albopunctata* |  | 40.393 | -7.601 | Portugal | Guarda, Seia, Vale do Rossim |
| INV05544 | Tettigoniinae | OR974442 | IBIOR195-22 | *Platycleis albopunctata* |  | 42.001 | -8.288 | Portugal | Viana do Castelo, Melgaco, Gave |
| TTT338 | Tettigoniinae | PQ568852 | TETTI392-24 | *Platycleis albopunctata* |  | 41.401 | -7.492 | Portugal | Vila Real, Murça, Fiolhoso |
| TTT264 | Tettigoniinae | PQ568841 | TETTI381-24 | *Platycleis albopunctata* |  | 40.421 | -5.298 | Spain | Ávila, Santiago del Collado, Puerto de la Peña Negra |
| TTT197 | Tettigoniinae | PQ568834 | TETTI374-24 | *Platycleis albopunctata* |  | 42.646 | -4.167 | Spain | Burgos, Amaya |
| JGR299 | Tettigoniinae | PQ568499 | TETTI038-24 | *Platycleis albopunctata* |  | 40.454 | -0.14 | Spain | Castellón, Ares del Maestre, Coll d'Ares |
| TTT165 | Tettigoniinae | PQ568828 | TETTI368-24 | *Platycleis albopunctata* |  | 39.887 | -2.818 | Spain | Cuenca, Segóbriga |
| TTT031 | Tettigoniinae | PQ568809 | TETTI349-24 | *Platycleis albopunctata* |  | 40.93 | -3.425 | Spain | Guadalajara, 3 km NO Alpedrete Sierra |
| TTT336 | Tettigoniinae | PQ568851 | TETTI391-24 | *Platycleis albopunctata* | # *Pl. intermedia* | 42.582 | 0.546 | Spain | Huesca, Benasque, Cerler |
| T090437 | Tettigoniinae | PQ568567 | TETTI106-24 | *Platycleis albopunctata* |  | 38.872 | 1.343 | Spain | Ibiza, Sant Josep |
| T090439 | Tettigoniinae | PQ568568 | TETTI107-24 | *Platycleis albopunctata* |  | 38.872 | 1.343 | Spain | Ibiza, Sant Josep |
| TTT191 | Tettigoniinae | PQ568833 | TETTI373-24 | *Platycleis albopunctata* |  | 42.925 | -5.869 | Spain | León, Caldas de Luna |
| TTT152 | Tettigoniinae | PQ568823 | TETTI363-24 | *Platycleis albopunctata* |  | 40.206 | -3.291 | Spain | Madrid, 4 km S Tielmes |
| TTT173 | Tettigoniinae | PQ568830 | TETTI370-24 | *Platycleis albopunctata* |  | 40.241 | -3.356 | Spain | Madrid, Perales de Tajuña |
| JGR245 | Tettigoniinae | PQ568492 | TETTI031-24 | *Platycleis albopunctata* |  | 40.57 | -4.208 | Spain | Madrid, Robledondo |
| TTT327 | Tettigoniinae | PQ568849 | TETTI389-24 | *Platycleis albopunctata* |  | 40.251 | -3.308 | Spain | Madrid, Tielmes |
| TTT179 | Tettigoniinae | PQ568831 | TETTI371-24 | *Platycleis albopunctata* |  | 40.075 | -3.248 | Spain | Madrid, Villamanrique de Tajo |
| TTT097 | Tettigoniinae | PQ568816 | TETTI356-24 | *Platycleis albopunctata* |  | 39.748 | -3.2 | Spain | Toledo, Corral de Almaguer |
| TTT098 | Tettigoniinae | PQ568817 | TETTI357-24 | *Platycleis albopunctata* |  | 39.748 | -3.2 | Spain | Toledo, Corral de Almaguer |
| TTT099 | Tettigoniinae | PQ568818 | TETTI358-24 | *Platycleis albopunctata* |  | 39.748 | -3.2 | Spain | Toledo, Corral de Almaguer |
| TTT269 | Tettigoniinae | PQ568842 | TETTI382-24 | *Platycleis albopunctata* |  | 42.035 | -6.922 | Spain | Zamora, Lubián-Chanos |
| **ID** | **Subfamily** | **Genbank** | **BOLD code** | **Species** | **Introgression Event** | **Latitude** | **Longitude** | **Country** | **Locality** |
| T090638 | Tettigoniinae | PQ568592 | TETTI131-24 | *Platycleis albopunctata maura* |  | 32.853 | -5.128 | Morocco | Midelt, Izert |
| T090637 | Tettigoniinae | PQ568591 | TETTI130-24 | *Platycleis albopunctata maura* |  | 32.467 | -5.306 | Morocco | Midelt, Tounfite |
| JGR069 | Tettigoniinae | PQ568466 | TETTI005-24 | *Platycleis falx* |  | 40.395 | -4.002 | Spain | Madrid, Brunete |
| JGR071 | Tettigoniinae | PQ568468 | TETTI007-24 | *Platycleis falx* |  | 40.395 | -4.002 | Spain | Madrid, Brunete |
| JGR173 | Tettigoniinae | PQ568477 | TETTI016-24 | *Platycleis falx* |  | 40.417 | -4.059 | Spain | Madrid, Quijorna |
| TTT217 | Tettigoniinae | PQ568839 | TETTI379-24 | *Platycleis falx* |  | 40.201 | -3.203 | Spain | Madrid, Valdaracete |
| T091044 | Tettigoniinae | PQ568663 | TETTI202-24 | *Platycleis iberica* |  | 40.302 | -5.179 | Spain | Ávila, Hoyo del Espino, Garganta de la Covacha |
| T092206 | Tettigoniinae | PQ568795 | TETTI335-24 | *Platycleis iberica* |  | 40.331 | -5.129 | Spain | Ávila, Navarredonda de Gredos, Valdeascas |
| T092207 | Tettigoniinae | PQ568796 | TETTI336-24 | *Platycleis iberica* |  | 40.331 | -5.129 | Spain | Ávila, Navarredonda de Gredos, Valdeascas |
| INV02518 | Tettigoniinae | OR974615 | IBIOR083-17 | *Platycleis intermedia* |  | 37.619 | -7.976 | Portugal | Beja, Castro Verde, Santa Barbara dos Padroes |
| INV10586 | Tettigoniinae | OR974728 | IBIOR532-22 | *Platycleis intermedia* |  | 37.653 | -7.644 | Portugal | Beja, Mertola, Bombeira do Guadiana |
| JGR239 | Tettigoniinae | PQ568490 | TETTI029-24 | *Platycleis intermedia* |  | 40.847 | -3.137 | Spain | Guadalajara, Humanes |
| JGR240 | Tettigoniinae | PQ568491 | TETTI030-24 | *Platycleis intermedia* |  | 40.847 | -3.137 | Spain | Guadalajara, Humanes |
| JGR064 | Tettigoniinae | PQ568465 | TETTI004-24 | *Platycleis intermedia* |  | 40.377 | -4.205 | Spain | Madrid, Chapineria |
| JGR251 | Tettigoniinae | PQ568494 | TETTI033-24 | *Platycleis intermedia* |  | 40.728 | -4.081 | Spain | Madrid, Los Molinos |
| JGR186 | Tettigoniinae | PQ568480 | TETTI019-24 | *Platycleis intermedia* |  | 40.423 | -3.379 | Spain | Madrid, Torres de la Alameda |
| JGR084 | Tettigoniinae | PQ568470 | TETTI009-24 | *Platycleis intermedia* |  | 40.473 | -3.609 | Spain | Madrid, Valdebebas |
| JGR262 | Tettigoniinae | PQ568496 | TETTI035-24 | *Platycleis intermedia* |  | 40.202 | -3.26 | Spain | Madrid, Villarejo de Salvanes |
| INV00132 | Tettigoniinae | OR974756 | IBIOR059-17 | *Platycleis sabulosa* | # *Pl. albopunctata* | 37.704 | -7.868 | Portugal | Beja, Mertola, Alcaria Ruiva |
| INV10593 | Tettigoniinae | OR974814 | IBIOR534-22 | *Platycleis sabulosa* |  | 37.653 | -7.644 | Portugal | Beja, Mertola, Bombeira do Guadiana |
| INV10326 | Tettigoniinae | OR974468 | IBIOR527-22 | *Platycleis sabulosa* | # *Pl. albopunctata* | 37.677 | -7.849 | Portugal | Beja, Mertola, Herdade de Alagaes |
| INV11434 | Tettigoniinae | OR974630 | IBIOR551-22 | *Platycleis sabulosa* | # *Pl. albopunctata* | 37.677 | -7.849 | Portugal | Beja, Mertola, Herdade de Alagaes |
| INV11452 | Tettigoniinae | OR974596 | IBIOR554-22 | *Platycleis sabulosa* | # *Pl. albopunctata* | 37.677 | -7.849 | Portugal | Beja, Mertola, Herdade de Alagaes |
| INV00133 | Tettigoniinae | OR974450 | IBIOR060-17 | *Platycleis sabulosa* | # *Pl. albopunctata* | 37.660 | -7.875 | Portugal | Beja, Mertola, Sao Joao dos Caldeireiros |
| INV00131 | Tettigoniinae | OR974479 | IBIOR058-17 | *Platycleis sabulosa* | # *Pl. albopunctata* | 37.757 | -8.234 | Portugal | Beja, Ourique, Conceicao |
| INV00134 | Tettigoniinae | OR974690 | IBIOR061-17 | *Platycleis sabulosa* | # *Pl albopunctata* | 37.635 | -8.170 | Portugal | Beja, Ourique, Ourique |
| INV05439 | Tettigoniinae | OR974658 | IBIOR399-22 | *Platycleis sabulosa* |  | 41.316 | -7.220 | Portugal | Bragança, Vila Flor, Freixiel |
| INV05515 | Tettigoniinae | OR974429 | IBIOR403-22 | *Platycleis sabulosa* | # *Pl. albopunctata* | 41.331 | -7.240 | Portugal | Bragança, Vila Flor, Freixiel |
| INV05516 | Tettigoniinae | OR974716 | IBIOR404-22 | *Platycleis sabulosa* |  | 41.331 | -7.240 | Portugal | Bragança, Vila Flor, Freixiel |
| INV07352 | Tettigoniinae | OR974679 | IBIOR468-22 | *Platycleis sabulosa* | # *Pl. albopunctata* | 41.332 | -7.257 | Portugal | Bragança, Vila Flor, Vieiro |
| INV07353 | Tettigoniinae | OR974671 | IBIOR469-22 | *Platycleis sabulosa* |  | 41.332 | -7.257 | Portugal | Bragança, Vila Flor, Vieiro |
| INV02571 | Tettigoniinae | OR974581 | IBIOR128-17 | *Platycleis sabulosa* | # *Pl. albopunctata* | 38.510 | -9.151 | Portugal | Setubal, Sesimbra, Pinhal da Aiana |
| TTT092 | Tettigoniinae | PQ568813 | TETTI353-24 | *Platycleis sabulosa* | # *Pl. albopunctata* | 39.329 | -3.363 | Spain | Ciudad Real, 4 km S Herencia |
| TTT111 | Tettigoniinae | PQ568819 | TETTI359-24 | *Platycleis sabulosa* |  | 39.518 | -2.871 | Spain | Cuenca, 2 km N Mota del Cuervo |
| **ID** | **Subfamily** | **Genbank** | **BOLD code** | **Species** | **Introgression Event** | **Latitude** | **Longitude** | **Country** | **Locality** |
| TTT146 | Tettigoniinae | PQ568820 | TETTI360-24 | *Platycleis sabulosa* | # *Pl. albopunctata* | 39.427 | -2.763 | Spain | Cuenca, 4 km N Las Mesas |
| TTT147 | Tettigoniinae | PQ568821 | TETTI361-24 | *Platycleis sabulosa* | # *Pl. albopunctata* | 39.427 | -2.763 | Spain | Cuenca, 4 km N Las Mesas |
| TTT148 | Tettigoniinae | PQ568822 | TETTI362-24 | *Platycleis sabulosa* |  | 39.427 | -2.763 | Spain | Cuenca, 4 km N Las Mesas |
| JGR233 | Tettigoniinae | PQ568487 | TETTI026-24 | *Platycleis sabulosa* | # *Pl. albopunctata* | 40.835 | -3.121 | Spain | Guadalajara, Alarilla |
| JGR234 | Tettigoniinae | PQ568488 | TETTI027-24 | *Platycleis sabulosa* | # *Pl. albopunctata* | 40.835 | -3.121 | Spain | Guadalajara, Alarilla |
| JGR235 | Tettigoniinae | PQ568489 | TETTI028-24 | *Platycleis sabulosa* | # *Pl. albopunctata* | 40.835 | -3.121 | Spain | Guadalajara, Alarilla |
| TTT044 | Tettigoniinae | PQ568811 | TETTI351-24 | *Platycleis sabulosa* | # *Pl. albopunctata* | 40.355 | -3.209 | Spain | Madrid, 2 km NE Villar del Olmo |
| TTT076 | Tettigoniinae | PQ568812 | TETTI352-24 | *Platycleis sabulosa* |  | 40.228 | -3.313 | Spain | Madrid, 2 km S Tielmes |
| JGR197 | Tettigoniinae | PQ568483 | TETTI022-24 | *Platycleis sabulosa* | # *Pl. albopunctata* | 40.379 | -4.005 | Spain | Madrid, Brunete |
| TTT209 | Tettigoniinae | PQ568838 | TETTI378-24 | *Platycleis sabulosa* | # *Pl. albopunctata* | - | - | Spain | Madrid, Casa de Campo |
| JGR103 | Tettigoniinae | PQ568471 | TETTI010-24 | *Platycleis sabulosa* | # *Pl. albopunctata* | 40.168 | -3.616 | Spain | Madrid, Ciempozuelos |
| JGR250 | Tettigoniinae | PQ568493 | TETTI032-24 | *Platycleis sabulosa* | # *Pl. albopunctata* | 40.728 | -4.081 | Spain | Madrid, Los Molinos |
| JGR199 | Tettigoniinae | PQ568484 | TETTI023-24 | *Platycleis sabulosa* |  | 40.371 | -4.015 | Spain | Madrid, Sevilla la Nueva |
| TTT199 | Tettigoniinae | PQ568835 | TETTI375-24 | *Platycleis sabulosa* |  | 40.154 | -3.3 | Spain | Madrid, SO Villarejo de Salvanés |
| TTT206 | Tettigoniinae | PQ568837 | TETTI377-24 | *Platycleis sabulosa* | # *Pl. albopunctata* | 40.154 | -3.3 | Spain | Madrid, SO Villarejo de Salvanés |
| TTT218 | Tettigoniinae | PQ568840 | TETTI380-24 | *Platycleis sabulosa* | # *Pl. albopunctata* | 40.222 | -3.203 | Spain | Madrid, Valdaracete |
| JGR104 | Tettigoniinae | PQ568472 | TETTI011-24 | *Platycleis sabulosa* | # *Pl. albopunctata* | 40.197 | -3.667 | Spain | Madrid, Valdemoro |
| JGR201 | Tettigoniinae | PQ568485 | TETTI024-24 | *Platycleis sabulosa* | # *Pl. albopunctata* | 40.346 | -4.108 | Spain | Madrid, Villanueva de Perales |
| JGR308 | Tettigoniinae | PQ568500 | TETTI039-24 | *Platycleis sabulosa* | # *P. albopunctata* | 37.932 | -5.747 | Spain | Sevilla, Cazalla de la Sierra |
| JGR309 | Tettigoniinae | PQ568501 | TETTI040-24 | *Platycleis sabulosa* |  | 37.932 | -5.747 | Spain | Sevilla, Cazalla de la Sierra |
| JGR310 | Tettigoniinae | PQ568502 | TETTI041-24 | *Platycleis sabulosa* | # *Pl. albopunctata* | 37.932 | -5.747 | Spain | Sevilla, Cazalla de la Sierra |
| TTT095 | Tettigoniinae | PQ568814 | TETTI354-24 | *Platycleis sabulosa* | # *Pl. albopunctata* | 39.748 | -3.2 | Spain | Toledo, Corral de Almaguer |
| TTT096 | Tettigoniinae | PQ568815 | TETTI355-24 | *Platycleis sabulosa* | # *Pl. albopunctata* | 39.748 | -3.2 | Spain | Toledo, Corral de Almaguer |
| TTT281 | Tettigoniinae | PQ568845 | TETTI385-24 | *Platycleis sabulosa* | # *Pl. albopunctata* | 41.738 | -6.171 | Spain | Zamora, Gallegos del Río |
| T091040 | Tettigoniinae | PQ568660 | TETTI199-24 | *Pterolepis cordubensis* |  | 37.353 | -5.584 | Spain | Sevilla, Carmona, Haza del Calderón |
| T091641 | Tettigoniinae | PQ568742 | TETTI281-24 | *Pterolepis cordubensis* |  | 37.475 | -6.088 | Spain | Sevilla, Salteras |
| INV06068 | Tettigoniinae | OR974774 | IBIOR410-22 | *Pterolepis grallata* |  | 41.206 | -7.403 | Portugal | Bragança, Carrazeda de Ansiaes, Quinta do Zimbro |
| INV06631 | Tettigoniinae | OR974566 | IBIOR434-22 | *Pterolepis grallata* |  | 41.349 | -7.290 | Portugal | Bragança, Mirandela, Abreiro |
| INV07351 | Tettigoniinae | OR974809 | IBIOR467-22 | *Pterolepis grallata* |  | 41.332 | -7.257 | Portugal | Bragança, Vila Flor, Vieiro |
| INV06609 | Tettigoniinae | OR974483 | IBIOR431-22 | *Pterolepis grallata* |  | 40.938 | -7.085 | Portugal | Guarda, Figueira de Castelo Rodrigo, Horta da Saboia - Faia Brava |
| INV07175 | Tettigoniinae | OR974830 | IBIOR570-22 | *Pterolepis grallata* |  | 41.284 | -7.396 | Portugal | Vila Real, Alijo, Amieiro |
| INV06069 | Tettigoniinae | OR974761 | IBIOR411-22 | *Pterolepis grallata* |  | 41.217 | -7.440 | Portugal | Vila Real, Alijo, Sao Mamede de Ribatua |
| T091202 | Tettigoniinae | PQ568678 | TETTI217-24 | *Pterolepis grallata* |  | 39.713 | -5.74 | Spain | Cáceres, Jaraicejo, Puerto de Miravete |
| T090388 | Tettigoniinae | PQ568556 | TETTI095-24 | *Pterolepis grallata* |  | 40.645 | -3.82 | Spain | Madrid, Colmenar Viejo, Ctra. Hoyo de Manzanares |
| **ID** | **Subfamily** | **Genbank** | **BOLD code** | **Species** | **Introgression Event** | **Latitude** | **Longitude** | **Country** | **Locality** |
| T090389 | Tettigoniinae | PQ568557 | TETTI096-24 | *Pterolepis grallata* |  | 40.645 | -3.82 | Spain | Madrid, Colmenar Viejo, Ctra. Hoyo de Manzanares |
| T090079 | Tettigoniinae | PQ568511 | TETTI050-24 | *Pterolepis grallata* |  | 40.154 | -3.337 | Spain | Madrid, Valdelaguna, La Tejera |
| T090102 | Tettigoniinae | PQ568515 | TETTI054-24 | *Pterolepis grallata* |  | 40.154 | -3.337 | Spain | Madrid, Valdelaguna, La Tejera |
| T090095 | Tettigoniinae | PQ568513 | TETTI052-24 | *Pterolepis grallata* |  | 40.163 | -3.34 | Spain | Madrid, Valdelaguna, La Vieja |
| INV07563 | Tettigoniinae | OR974437 | IBIOR479-22 | *Pterolepis lusitanica* |  | 37.193 | -7.921 | Portugal | Faro, Sao Bras de Alportel |
| T091265 | Tettigoniinae | PQ568685 | TETTI224-24 | *Pterolepis lusitanica* |  | 39.5 | -8.827 | Portugal | Leiria, Mendiga |
| T091269 | Tettigoniinae | PQ568686 | TETTI225-24 | *Pterolepis lusitanica* |  | 39.5 | -8.827 | Portugal | Leiria, Mendiga |
| INV05574 | Tettigoniinae | OR974551 | IBIOR225-22 | *Pterolepis lusitanica* |  | 39.008 | -8.452 | Portugal | Santarem, Coruche, Herdade dos Concelhos |
| T091683 | Tettigoniinae | PQ568754 | TETTI293-24 | *Pterolepis lusitanica* |  | 38.12 | -8.787 | Portugal | Setúbal, Santiago do Cacém |
| INV06945 | Tettigoniinae | OR974831 | IBIOR450-22 | *Pterolepis spoliata* | # *Pt. lusitanica* | 37.653 | -7.644 | Portugal | Beja, Mertola, Bombeira do Guadiana |
| INV08090 | Tettigoniinae | OR974424 | IBIOR241-22 | *Pterolepis spoliata* | # *Pt. lusitanica* | 38.126 | -8.789 | Portugal | Setubal, Grandola, Melides |
| T090313 | Tettigoniinae | PQ568535 | TETTI074-24 | *Pterolepis spoliata* |  | 36.928 | -2.292 | Spain | Almeria, Nijar, Cuevas de los Ubedas |
| T090166 | Tettigoniinae | PQ568525 | TETTI064-24 | *Pterolepis spoliata* |  | 36.925 | -3.29 | Spain | Granada, 3 km al N de Almegíjar |
| T090167 | Tettigoniinae | PQ568526 | TETTI065-24 | *Pterolepis spoliata* |  | 36.925 | -3.29 | Spain | Granada, 3 km al N de Almegíjar |
| T090168 | Tettigoniinae | PQ568527 | TETTI066-24 | *Pterolepis spoliata* |  | 36.925 | -3.29 | Spain | Granada, 3 km al N de Almegíjar |
| T090685 | Tettigoniinae | PQ568605 | TETTI144-24 | *Pterolepis spoliata* |  | 37.606 | -4.035 | Spain | Jaén, Alcaudete, Sierra Ahillo |
| T090028 | Tettigoniinae | PQ568506 | TETTI045-24 | *Pterolepis spoliata* | # *Pt. lusitanica* | 37.905 | -5.807 | Spain | Sevilla, Cazalla de la Sierra, Ctra. de las Colonias |
| T090029 | Tettigoniinae | PQ568507 | TETTI046-24 | *Pterolepis spoliata* | # *Pt. lusitanica* | 37.905 | -5.807 | Spain | Sevilla, Cazalla de la Sierra, Ctra. de las Colonias |
| T091344 | Tettigoniinae | PQ568696 | TETTI235-24 | *Roeseliana oporina* |  | 39.863 | -2.705 | Spain | Cuenca, Montalbo, Laguna de El Hito |
| T091091 | Tettigoniinae | PQ568671 | TETTI210-24 | *Roeseliana oporina* |  | 39.888 | -2.773 | Spain | Cuenca, Saelices, rivera del Gigüela |
| TTT375 | Tettigoniinae | PQ568854 | TETTI394-24 | *Roeseliana roeselii* |  | 42.823 | 0.798 | Spain | Lleida, San Juan de Toran |
| TTT376 | Tettigoniinae | PQ568855 | TETTI395-24 | *Roeseliana roeselii* |  | 42.823 | 0.798 | Spain | Lleida, San Juan de Toran |
| T090918 | Tettigoniinae | PQ568653 | TETTI192-24 | *Sepiana sepium* |  | 41.665 | 1.988 | Spain | Barcelona, Mura, Parque Natural de Sant Llorenç del Munt i l'Obac |
| T090054 | Tettigoniinae | PQ568510 | TETTI049-24 | *Sepiana sepium* |  | 41.852 | 2.671 | Spain | Girona, Santa Coloma de Farners |
| INV00137 | Tettigoniinae | OR974526 | IBIOR063-17 | *Tessellana tessellata* |  | 37.812 | -7.966 | Portugal | Beja, Castro Verde, Entradas |
| INV00136 | Tettigoniinae | OR974473 | IBIOR062-17 | *Tessellana tessellata* |  | 37.660 | -7.875 | Portugal | Beja, Mertola, Sao Joao dos Caldeireiros |
| INV06544 | Tettigoniinae | OR974556 | IBIOR422-22 | *Tessellana tessellata* |  | 41.332 | -7.257 | Portugal | Bragança, Vila Flor, Vieiro |
| INV06545 | Tettigoniinae | OR974522 | IBIOR423-22 | *Tessellana tessellata* |  | 41.332 | -7.257 | Portugal | Bragança, Vila Flor, Vieiro |
| INV03387 | Tettigoniinae | OR974616 | IBIOR154-19 | *Tessellana tessellata* |  | 40.385 | -7.617 | Portugal | Guarda, Gouveia, Near Vale Rossim |
| INV09723 | Tettigoniinae | OR974718 | IBIOR583-22 | *Tessellana tessellata* |  | 41.882 | -7.729 | Portugal | Vila Real, Montalegre, Serra do Larouco |
| JGR008 | Tettigoniinae | PQ568462 | TETTI001-24 | *Tessellana tessellata* |  | 40.873 | -3.595 | Spain | Madrid, La Cabrera |
| JGR177 | Tettigoniinae | PQ568479 | TETTI018-24 | *Tessellana tessellata* |  | 40.551 | -3.549 | Spain | Madrid, Paracuellos de Jarama |
| JGR140 | Tettigoniinae | PQ568473 | TETTI012-24 | *Tessellana tessellata* |  | 40.358 | -3.554 | Spain | Madrid, Rivas urbanización |
| JGR187 | Tettigoniinae | PQ568481 | TETTI020-24 | *Tessellana tessellata* |  | 40.423 | -3.379 | Spain | Madrid, Torres de la Alameda |
| **ID** | **Subfamily** | **Genbank** | **BOLD code** | **Species** | **Introgression Event** | **Latitude** | **Longitude** | **Country** | **Locality** |
| JGR166 | Tettigoniinae | PQ568474 | TETTI013-24 | *Tessellana tessellata* |  | 40.44 | -4.018 | Spain | Madrid, Villanueva de la Cañada |
| JGR260 | Tettigoniinae | PQ568495 | TETTI034-24 | *Tessellana tessellata* |  | 40.202 | -3.283 | Spain | Madrid, Villarejo de Salvanes |
| JGR056 | Tettigoniinae | PQ568464 | TETTI003-24 | *Tessellana tessellata* |  | 40.349 | -3.918 | Spain | Madrid, Villaviciosa de Odón |
| T090846 | Tettigoniinae | PQ568642 | TETTI181-24 | *Tettigonia cantans* |  | 42.679 | 0.706 | Spain | Lleida, Artiga de Lin |
| T090847 | Tettigoniinae | PQ568643 | TETTI182-24 | *Tettigonia cantans* |  | 42.769 | 0.662 | Spain | Lleida, Bossot, Puerto de Portillón |
| T090630 | Tettigoniinae | PQ568590 | TETTI129-24 | *Tettigonia hispanica* |  | 40.987 | -3.814 | Spain | Madrid, Lozoya, Puerto de Navafría |
| INV02524 | Tettigoniinae | OR974547 | IBIOR089-17 | *Tettigonia viridissima* |  | 37.619 | -7.976 | Portugal | Beja, Castro Verde, Santa Barbara dos Padroes |
| INV02525 | Tettigoniinae | OR974531 | IBIOR090-17 | *Tettigonia viridissima* |  | 37.620 | -7.976 | Portugal | Beja, Castro Verde, Santa Barbara dos Padroes |
| T091038 | Tettigoniinae | PQ568658 | TETTI197-24 | *Tettigonia viridissima* |  | 37.603 | -8.653 | Portugal | Beja, Odemira |
| INV05451 | Tettigoniinae | OR974520 | IBIOR401-22 | *Tettigonia viridissima* |  | 41.315 | -7.220 | Portugal | Bragança, Vila Flor, Freixiel |
| T090461 | Tettigoniinae | PQ568570 | TETTI109-24 | *Tettigonia viridissima* |  | 42.882 | -7.704 | Spain | Lugo, Guntín de Pallares |
| INV05307 | Tettigoniinae | OR974597 | IBIOR396-22 | *Thyreonotus bidens* |  | 41.287 | -7.383 | Portugal | Bragança, Carrazeda de Ansiaes, Quinta de Barrabaz |
| INV04118 | Tettigoniinae | OR974598 | IBIOR359-22 | *Thyreonotus bidens* |  | 41.057 | -6.906 | Portugal | Bragança, Freixo de Espada a Cinta, Ribeira do Mosteiro |
| INV07109 | Tettigoniinae | OR974499 | IBIOR457-22 | *Thyreonotus bidens* |  | 41.315 | -7.220 | Portugal | Bragança, Vila Flor, Freixiel |
| INV07232 | Tettigoniinae | OR974725 | IBIOR462-22 | *Thyreonotus bidens* |  | 41.332 | -7.257 | Portugal | Bragança, Vila Flor, Vieiro |
| INV06570 | Tettigoniinae | OR974439 | IBIOR427-22 | *Thyreonotus bidens* |  | 41.374 | -7.203 | Portugal | Bragança, Vila Flor, Vilarinho das Azenhas |
| INV07796 | Tettigoniinae | OR974500 | IBIOR490-22 | *Thyreonotus bidens* |  | 37.305 | -8.553 | Portugal | Faro, Monchique, Monchique |
| INV02581 | Tettigoniinae | OR974766 | IBIOR137-17 | *Thyreonotus bidens* |  | 38.547 | -9.045 | Portugal | Setubal, Sesimbra, Cabeco Melao |
| INV07805 | Tettigoniinae | OR974471 | IBIOR491-22 | *Thyreonotus bidens* |  | 38.475 | -8.994 | Portugal | Setubal, Setubal, Convento da Arrabida |
| T090385 | Tettigoniinae | PQ568555 | TETTI094-24 | *Thyreonotus bidens* |  | 40.645 | -3.82 | Spain | Madrid, Colmenar Viejo, Ctra.Hoyo de Manzanares |
| T090551 | Tettigoniinae | PQ568587 | TETTI126-24 | *Thyreonotus bidens* |  | 37.971 | -5.664 | Spain | Sevilla, San Nicolas del Puerto |
| T091054 | Tettigoniinae | PQ568667 | TETTI206-24 | *Thyreonotus corsicus* |  | 36.859 | -5.392 | Spain | Cádiz, Algodonales |
| T090392 | Tettigoniinae | PQ568559 | TETTI098-24 | *Thyreonotus corsicus* |  | 40.336 | -1.761 | Spain | Cuenca, Tragacete |
| T090393 | Tettigoniinae | PQ568560 | TETTI099-24 | *Thyreonotus corsicus* |  | 40.336 | -1.761 | Spain | Cuenca, Tragacete |
| T090140 | Tettigoniinae | PQ568516 | TETTI055-24 | *Yersinella raymondii* |  | 39.934 | 9.453 | Italy | Sardegna, Nuoro Villagrande Strisaili |
| T090048 | Tettigoniinae | PQ568508 | TETTI047-24 | *Yersinella raymondii* |  | 41.852 | 2.671 | Spain | Girona, Santa Coloma de Farners |
| T090052 | Tettigoniinae | PQ568509 | TETTI048-24 | *Yersinella raymondii* |  | 41.852 | 2.671 | Spain | Girona, Santa Coloma de Farners |
| T090678 | Tettigoniinae | PQ568603 | TETTI142-24 | *Zeuneriana abbreviata* |  | 42.971 | -1.008 | Spain | Navarra, Larraun, Selva de Irati |
| T090679 | Tettigoniinae | PQ568604 | TETTI143-24 | *Zeuneriana abbreviata* |  | 42.971 | -1.008 | Spain | Navarra, Larraun, Selva de Irati |
| T090347 | Tettigoniinae | PQ568545 | TETTI084-24 | *Zeuneriana burriana* |  | 43.232 | -4.811 | Spain | Asturias, Cabrales, Bulnes |
| T090348 | Tettigoniinae | PQ568546 | TETTI085-24 | *Zeuneriana burriana* |  | 43.232 | -4.811 | Spain | Asturias, Cabrales, Bulnes |
| T090349 | Tettigoniinae | PQ568547 | TETTI086-24 | *Zeuneriana burriana* |  | 43.232 | -4.811 | Spain | Asturias, Cabrales, Bulnes |

Table S2. Mean and maximum intraspecific genetic distances and number of sequences included for each species.

|  | **Species** | **Nº samples** | **Mean distance** | **Max distance** |  |  | **Species** | | **Nº samples** | **Mean distance** | **Max distance** |
| --- | --- | --- | --- | --- | --- | --- | --- | --- | --- | --- | --- |
|  |  |  |  |  |  |  |  |  |  |  |  |
| **Bradyporinae** | |  |  |  |  | **Meconematinae** | |  | |  |  |
|  | *Albarracinia zapaterii* | 2 | 0.015 | 0.015 |  |  | *Canariola emarginata* | | 1 | NA | NA |
|  | *Baetica ustulata* | 2 | 0.013 | 0.013 |  |  | *Cyrtaspis scutata* | | 4 | 0.002 | 0.003 |
|  | *Baratia sari* | 2 | 0 | 0 |  |  | *Cyrtaspis tuberculata* | | 1 | NA | NA |
|  | *Callicrania demandae* | 2 | 0.002 | 0.002 |  |  | *Meconema thalassinum* | | 4 | 0.001 | 0.002 |
|  | *Callicrania faberi* | 1 | NA | NA |  | **Phaneropterinae** | |  | |  |  |
|  | *Callicrania plaxicauda* | 2 | 0.005 | 0.005 |  |  | *Barbitistes fischeri* | | 3 | 0.003 | 0.005 |
|  | *Callicrania ramburii* | 1 | NA | NA |  |  | *Isophya pyrenaea* | | 1 | NA | NA |
|  | *Callicrania vicentae* | 1 | NA | NA |  |  | *Leptophyes punctatissima* | | 3 | 0.003 | 0.005 |
|  | *Coracinotus notaroi* | 3 | 0.037 | 0.056 |  |  | *Metaplastes pulchripennis* | | 2 | 0.002 | 0.002 |
|  | *Coracinotus politus* | 1 | NA | NA |  |  | *Odontura aspericauda* | | 3 | 0.047 | 0.053 |
|  | *Ephippiger diurnus* | 3 | 0.012 | 0.018 |  |  | *Odontura glabricauda* | | 8 | 0.003 | 0.006 |
|  | *Ephippigerida areolaria* | 1 | NA | NA |  |  | *Odontura macphersoni* | | 4 | 0.008 | 0.014 |
|  | *Ephippigerida* aff. *areolaria* | 2 | NA | NA |  |  | *Phaneroptera falcata* | | 2 | 0.003 | 0.003 |
|  | *Ephippigerida asella* | 2 | 0 | 0 |  |  | *Phaneroptera nana* | | 21 | 0.004 | 0.01 |
|  | *Ephippigerida barati* | 1 | NA | NA |  |  | *Phaneroptera sparsa* | | 4 | 0 | 0 |
|  | *Ephippigerida carinata* | 3 | 0.008 | 0.012 |  |  | *Tylopsis lilifolia* | | 8 | 0.001 | 0.003 |
|  | *Ephippigerida diluta* | 3 | 0.003 | 0.005 |  | **Saginae** | |  | |  |  |
|  | *Ephippigerida* aff. *diluta* 1 | 1 | NA | NA |  |  | *Saga pedo* | | 2 | 0 | 0 |
|  | *Ephippigerida* aff. *diluta* 2 | 2 | 0.008 | 0.008 |  | **Tettigoniinae** | |  | |  |  |
|  | *Ephippigerida fernandezi* | 1 | NA | NA |  |  | *Amphiestris baetica* | | 2 | 0 | 0 |
|  | *Ephippigerida laserena* | 1 | 0.007 | 0.007 |  |  | *Antaxius chopardi* | | 2 | 0.007 | 0.007 |
|  | *Ephippigerida pantingana* | 1 | NA | NA |  |  | *Antaxius florezi* | | 12 | 0.023 | 0.038 |
|  | *Ephippigerida rosae* | 3 | 0.009 | 0.012 |  |  | *Antaxius hispanicus* | | 5 | 0.005 | 0.012 |
|  | *Ephippigerida saussuriana* | 1 | NA | NA |  |  | *Antaxius kraussii* | | 5 | 0.001 | 0.003 |
|  | *Lluciapomaresius anapaulae* | 4 | 0.008 | 0.013 |  |  | *Antaxius oretanus* | | 8 | 0.031 | 0.054 |
|  | *Lluciapomaresius asturiensis* | 5 | 0.009 | 0.015 |  |  | *Antaxius spinibrachius* | | 27 | 0.024 | 0.045 |
|  | *Lluciapomaresius eclipticus* | 1 | NA | NA |  |  | *Bicolorana bicolor* | | 2 | 0.021 | 0.021 |
|  | *Lluciapomaresius nobrei* | 5 | 0.002 | 0.003 |  |  | *Ctenodecticus granatensis* | | 4 | 0.003 | 0.003 |
|  | *Lluciapomaresius ortegai* | 2 | 0 | 0 |  |  | *Ctenodecticus lusitanicus* | | 3 | 0.002 | 0.003 |
|  | *Lluciapomaresius panteli* | 3 | 0.005 | 0.008 |  |  | *Ctenodecticus major* | | 2 | 0.002 | 0.002 |
|  | *Lluciapomaresius stalii* | 3 | 0.026 | 0.037 |  |  | *Ctenodecticus masferreri* | | 2 | 0.005 | 0.005 |
|  | *Neocallicrania barrosi* | 4 | 0.054 | 0.084 |  |  | *Ctenodecticus pupulus* | | 3 | 0.01 | 0.015 |
|  | *Neocallicrania bolivarii* | 3 | 0.024 | 0.03 |  |  | *Ctenodecticus ramburi* | | 4 | 0.004 | 0.005 |
|  | *Neocallicrania lusitanica* | 7 | 0.034 | 0.06 |  |  | *Ctenodecticus thymi* | | 2 | 0 | 0 |
|  | *Neocallicrania miegii* | 5 | 0.003 | 0.005 |  |  | *Decticus albifrons* | | 5 | 0.007 | 0.015 |
|  | *Neocallicrania selligera* | 9 | 0.049 | 0.071 |  |  | *Decticus verrucivorus* | | 2 | 0.005 | 0.005 |
|  | *Neocallicrania serrata* | 5 | 0.041 | 0.071 |  |  | *Incertana decorata* | | 6 | 0.003 | 0.005 |
|  | *Parasteropleurus perezii* | 2 | 0.091 | 0.091 |  |  | *Metrioptera ambigua* | | 4 | 0.003 | 0.007 |
|  | *Platystolus martinezii* | 4 | 0.049 | 0.08 |  |  | *Metrioptera buyssoni* | | 3 | 0.003 | 0.005 |
|  | *Platystolus surcularius* | 2 | 0.018 | 0.018 |  |  | *Metrioptera maritima* | | 3 | 0.011 | 0.014 |
|  | *Pycnogaster algecirensis* | 1 | NA | NA |  |  | *Metrioptera saussuriana* | | 7 | 0.002 | 0.003 |
|  | *Pycnogaster cucullatus* | 3 | 0.039 | 0.039 |  |  | *Montana carpetana* | | 3 | 0.007 | 0.008 |
|  | *Pycnogaster gaditana* | 1 | NA | NA |  |  | *Pholidoptera griseoaptera* | | 3 | 0.001 | 0.002 |
|  | *Pycnogaster graellsii* | 3 | 0.021 | 0.03 |  |  | *Platycleis affinis* | | 10 | 0.006 | 0.014 |
|  | *Pycnogaster inermis* | 10 | 0.017 | 0.03 |  |  | *Platycleis albopunctata* | | 26 | 0.015 | 0.035 |
|  | *Pycnogaster jugicola* | 20 | 0.019 | 0.053 |  |  | *Platycleis falx* | | 4 | 0.012 | 0.019 |
|  | *Pycnogaster sanchezgomezi* | 3 | 0.027 | 0.022 |  |  | *Platycleis iberica* | | 3 | 0.002 | 0.003 |
|  | *Pycnogaster* sp. nov. 1 - Algarve | 2 | 0.002 | 0.002 |  |  | *Platycleis intermedia* | | 9 | 0.005 | 0.008 |
|  | *Pycnogaster* rosae | 1 | NA | NA |  |  | *Platycleis sabulosa* | | 40 | 0.003 | 0.008 |
|  | *Pycnogaster valentini* | 9 | 0.01 | 0.015 |  |  | *Pterolepis cordubensis* | | 2 | 0.007 | 0.007 |
|  | *Sabaterpia taeniata* | 1 | NA | NA |  |  | *Pterolepis grallata* | | 12 | 0.007 | 0.017 |
|  | *Sorapagus catalaunicus* | 4 | 0.018 | 0.027 |  |  | *Pterolepis lusitanica* | | 5 | 0.009 | 0.012 |
|  | *Steropleurus andalusicus* | 4 | 0.068 | 0.095 |  |  | *Pterolepis spoliata* | | 9 | 0.018 | 0.024 |
|  | *Steropleurus brunnerii* | 4 | 0.018 | 0.028 |  |  | *Roeseliana oporina* | | 2 | 0 | 0 |
|  | *Steropleurus flavovittatus* | 3 | 0.026 | 0.035 |  |  | *Roeseliana roeselii* | | 2 | 0.007 | 0.007 |
|  | *Steropleurus pseudolus* | 2 | 0.032 | 0.032 |  |  | *Sepiana sepium* | | 2 | 0 | 0 |
|  | *Steropleurus* aff. *pseudolus* | 2 | 0 | 0 |  |  | *Tessellana tessellata* | | 13 | 0.018 | 0.038 |
|  | *Steropleurus recticarinatus* | 2 | 0.002 | 0.002 |  |  | *Tettigonia cantans* | | 2 | 0 | 0 |
|  | *Steropleurus* sp. nov. 1 - Sª Nevada | 1 | NA | NA |  |  | *Tettigonia hispanica* | | 1 | NA | NA |
|  | *Steropleurus* sp. nov. 2 - Algarve | 2 | 0.007 | 0.007 |  |  | *Tettigonia viridissima* | | 5 | 0.01 | 0.015 |
|  | *Synephippius obvius* | 2 | 0.007 | 0.007 |  |  | *Thyreonotus bidens* | | 10 | 0.019 | 0.045 |
|  | *Uromenus agarenus* | 1 | NA | NA |  |  | *Thyreonotus corsicus* | | 3 | 0.023 | 0.035 |
|  | *Uromenus brevicollis* | 1 | NA | NA |  |  | *Yersinella raymondii* | | 3 | 0.001 | 0.002 |
|  | *Uromenus maroccanus* | 1 | NA | NA |  |  | *Zeuneriana abbreviata* | | 2 | 0.003 | 0.003 |
|  | *Uromenus rugosicollis* | 2 | 0 | 0 |  |  | *Zeuneriana burriana* | | 3 | 0 | 0 |
| **Conocephalinae** | |  |  |  |  |  |  | |  |  |  |
|  | *Conocephalus conocephalus* | 8 | 0.002 | 0.005 |  |  |  | |  |  |  |
|  | *Conocephalus fuscus* | 8 | 0.005 | 0.011 |  |  |  | |  |  |  |
|  | *Ruspolia nitidula* | 6 | 0.005 | 0.013 |  |  |  | |  |  |  |

Figure S1. ML phylogram of Conocephalinae species using IQTREE2. Values above branches indicate bootstrap support values.

Figure S2. ML phylogram of Meconematinae species using IQTREE2. Values above branches indicate bootstrap support values.

Figure S3. ML phylogram of Phaneropterinae species using IQTREE2. Values above branches indicate bootstrap support values.

Figure S4. Intra- and intergeneric genetic distances within each subfamily.


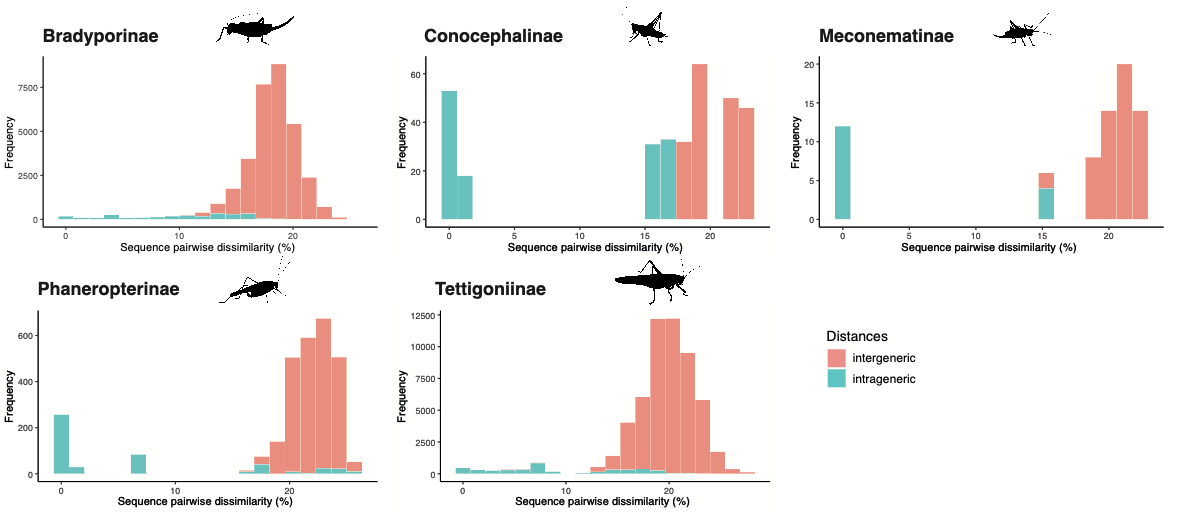

Supplement: Supplementary file 1 — Supplementary Material 1 [file 41598_2025_6695_MOESM1_ESM.docx]
